# Accelerated biological aging, healthy behaviors, and genetic susceptibility with incidence of stroke and its subtypes: A prospective cohort study

**DOI:** 10.1111/acel.14427

**Published:** 2024-12-04

**Authors:** Xuening Zhang, Hao Zhao, Zilin Li, Xinjie Liu, Yurong Zhang, Ning Yang, Tongchao Zhang, Xiaorong Yang, Ming Lu

**Affiliations:** ^1^ Clinical Epidemiology Unit Qilu Hospital of Shandong University Jinan Shandong China; ^2^ Department of Epidemiology, School of Public Health, Qilu Hospital, Cheeloo College of Medicine Shandong University Jinan Shandong China; ^3^ Clinical Research Center of Shandong University, Qilu Hospital, Cheeloo College of Medicine Shandong University Jinan Shandong China; ^4^ Department of Medical Statistics and Epidemiology, School of Public Health Sun Yat‐Sen University Guangzhou Guangdong China; ^5^ Department of Neurosurgery, Qilu Hospital, Cheeloo College of Medicine, Institute of Brain and Brain‐Inspired Science Shandong University Jinan China

**Keywords:** biological age acceleration, genetic susceptibility, healthy behaviors, prospective study, stroke

## Abstract

Stroke risk increases with chronological age, but the relationship with biological age (BA) acceleration is poorly understood. We aimed to examine the association between BA acceleration and incident stroke and its subtypes, explore the modifying effects on genetic susceptibility, and assess how BA acceleration mediates the effect of behavior score. We studied 253,932 UK Biobank participants and computed two BA measures (Klemera‐Doubal Method [KDM], Phenotypic Age [PhenoAge]), with BA acceleration calculated by regressing BA on chronological age. The polygenic risk score (PRS) was derived from 87 genetic loci. The behaviors score was based on diet, physical activity, tobacco/nicotine, sleep, and BMI. During a median follow‐up of 13.6 years, 5460 strokes, 4337 ischemic stroke (IS), 951 intracerebral hemorrhage (ICH), and 553 subarachnoid hemorrhage (SAH) cases were documented. Adjusting for confounding factors, each standard deviation increase in BA acceleration was associated with higher stroke risk: for KDM‐BA acceleration, stroke (HR = 1.28, 95% CI = 1.25–1.32), IS (1.32, 1.28–1.36), ICH (1.15, 1.08–1.23), and SAH (1.16, 1.07–1.27); for PhenoAge acceleration, stroke (1.22, 1.19–1.25), IS (1.26, 1.22–1.29), ICH (1.08, 1.02–1.16), and SAH (1.08, 1.00–1.18). Compared to participants with the lowest PRS and BA acceleration, those with the highest PRS and BA acceleration had the highest stroke risk (KDM‐BA acceleration: 2.19, 1.85–2.59; PhenoAge acceleration: 2.03, 1.69–2.42). Additionally, there was an additive interaction between KDM‐BA acceleration and PRS. The mediation proportion of BA acceleration in associations of behaviors score with incident stroke and its subtypes ranged from 15.84% to 33.08%. BA acceleration may raise stroke risk, especially in those with high genetic risk. Maintaining healthy behaviors may help mitigate this risk.

## Graphical abstract

This study identifies a significant association between accelerated biological aging, measured by clinical biomarker‐based KDM‐BA and PhenoAge accelerations, and an increased risk of stroke and its subtypes. The combination of accelerated biological aging with high genetic risk helps identify high‐risk populations on stroke development. Adopting the five healthy behaviors may slow biological aging and reduce stroke risk.

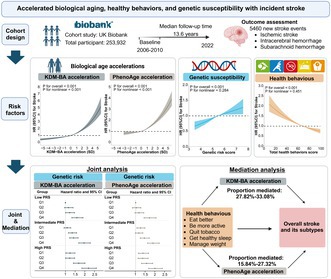

**Abbreviations**
AHA: American Heart Association
AP: attributable proportion
BA: biological age
BMI: body mass index
CI: confidence interval
DALYs: disability‐adjusted life years
DE: direct effect
FEV1: forced expiratory volume in 1‐second
GWAS: genome‐wide association study
HR: hazard ratio
ICD: International Classification of Disease
ICH: intracerebral hemorrhage
IE: indirect effects
IMD: Indices of Multiple Deprivation
IS: ischemic stroke
KDM: Klemera‐Doubal Method
KDM‐BA: Klemera‐Doubal Method Biological Age
NHANES: National Health and Nutrition Examination Surveys
NHS: National Health Service
Phenotypic Age: PhenoAge
PRS: polygenic risk score
Q: Quartile
RERI: relative excess risk due to interaction
SAH: subarachnoid hemorrhage
SBP: systolic blood pressure
SD: standard deviation
TE: total effect

## 1 INTRODUCTION

Stroke is one of the leading causes of death and loss of disability‐adjusted life years (DALYs) worldwide (GBD 2019 Demographics Collaborators, 2020). According to the 2019 Global Burden of Disease (GBD) study, there were 12.2 million new cases of stroke, resulting in 143 million DALYs and 6.55 million deaths (GBD 2019 Stroke Collaborators, 2021). Identifying the risk factors and mechanisms of stroke is a public health priority. Although chronological age is one of the foremost risk factors for stroke, there is significant heterogeneity in the aging trajectories among individuals with the same chronological age (Lowsky et al., 2014). Biological age (BA), incorporating information from biomarkers, provides a better reflection of an individual's physiological condition and the risk of age‐related diseases and mortality (Jylhävä et al., 2017).

Biological aging is a multi‐stage biological process that gradually injures the integrity and regenerative capabilities of cells, tissues, and organs (Kennedy et al., 2014). To comprehensively assess the state of biological aging, several approaches have been proposed, ranging from single biomarkers such as telomere length to the integrated analysis of encompassing epigenetics, leukocyte telomere length, and retinal age (D'Mello et al., 2015; Soriano‐Tárraga et al., 2016; Waziry et al., 2023; Zhang et al., 2023; Zhu et al., 2022). In addition, various single clinical indices such as cholesterol, glucose, blood pressure (Kim et al., 2018; Lee et al., 2018), and glycosylated hemoglobin (Au Yeung et al., 2018) have been proven to be associated with stroke risk. Recently, the BA algorithm that integrates multiple clinical indicators has been demonstrated to be one of the most precise and clinically practical methods for predicting both incidence and mortality rates (Belsky et al., 2018; Li et al., 2020). Klemera‐Doubal Method Biological Age (KDM‐BA) models BA as the average biological state associated with a specific chronological age within the reference population, with the assumption that BA increases linearly over time (Klemera & Doubal, 2006). Phenotypic Age (PhenoAge) models BA as the average biological state associated with a specific level of mortality risk within the reference population, with the assumption that BA increases exponentially over time (Liu et al., 2018). We calculated BA values for participants using KDM‐BA and the PhenoAge algorithm (Klemera & Doubal, 2006; Levine, 2013), both of which have been validated in multi‐ethnic cohorts to better predict disease, disability, and mortality (Graf et al., 2022; Liu et al., 2018). BA acceleration was calculated as a residual from a linear regression of BA against chronological age, with a higher value indicating the acceleration of aging. However, the association between BA acceleration and the risk of stroke remains lacking.

Recent genome‐wide association studies (GWAS) have identified genetic loci associated with stroke (Malik et al., 2018), and polygenic risk score (PRS) based on these findings have proven to be effective in quantifying genetic risk for stroke (Kim et al., 2021; Rutten‐Jacobs et al., 2018). Previous research has typically explored the risks of stroke related to either acquired exposures or genetic variations in isolation. However, it is now widely recognized that acquired factors can modify the impact of genetic predisposition on disease. Therefore, studying the combined and interactive effects of BA acceleration and PRS is essential for precise stroke prediction and targeted interventions for high‐risk populations. Additionally, addressing BA acceleration is a critical challenge in the context of global aging. The American Heart Association (AHA) recently updated the cardiovascular health indicators, which include five relatively modifiable healthy behaviors: eating better, being more active, quitting tobacco, getting healthy sleep, and managing weight (Lloyd‐Jones et al., 2022). However, it remains unclear whether adherence to these behaviors can reduce stroke risk and to what extent this reduction is mediated by mitigating BA acceleration.

In this study, we aimed to construct two BA acceleration measures (KDM‐BA acceleration and PhenoAge acceleration) using data from a prospective cohort in the UK Biobank and evaluate their association with the risk of incident stroke. Additionally, we explored the combined effects and interactions between BA acceleration and PRS concerning incident stroke. Finally, we assessed the extent to which BA acceleration mediates the impact of behavior scores on incident stroke.

## 2 METHODS

### 2.1 Study design and population

This study is derived from the UK Biobank, which is a population‐based prospective cohort study (Sudlow et al., 2015). In brief, this cohort recruited over half a million participants aged 40–69 years from 22 assessment centers across England, Scotland, and Wales at baseline between 2006 and 2010 with multiple follow‐ups. At baseline, participants were asked to provide socio‐demographic factors, behavioral factors, and health information. Blood samples were collected for genotyping and biochemistry tests. The UK Biobank study was approved by the North West Multicenter Research Ethical Committee. All participants provided written informed consent.

Among the 502,336 participants with available data in the current study, those with a history of stroke or ischemic heart disease at baseline (*N* = 33,774) were excluded. We then excluded participants with missing data for BA (*N* = 153,494) and behavioral factors (*N* = 24,216). In addition, participants without genetic information, those with a mismatch between genetic sex and reported sex, or those of non‐white ethnicity (*N* = 36,824) were further excluded. Moreover, participants with KDM‐BA or PhenoAge values exceeding the mean ± 5 SD range (*N* = 96) were considered extreme outliers and consequently were excluded from the analysis to ensure the robustness of the study findings. Finally, 253,932 participants were involved in the current study. The flow diagram for the inclusion of participants is presented in Figure S1.

### 2.2 Assessment of biological age and biological age accelerations

We determined BA utilizing the best‐validated algorithms that could be implemented with data available in the UK Biobank: the KDM‐BA and PhenoAge algorithms, both of which are based on clinical and blood chemistry‐derived metrics. Detailed calculations and interpretations of these two measures have been previously comprehensively summarized (Gao et al., 2023; Graf et al., 2022; Kwon & Belsky, 2021; Mak et al., 2023). In brief, KDM‐BA was calculated through a series of regression analyses on biomarkers against chronological age and could be interpreted as the age at which the average physiology in the US National Health and Nutrition Examination Surveys (NHANES) III (i.e., the training sample) matches the physiology of an individual (Klemera & Doubal, 2006). PhenoAge was calculated using a mortality prediction score derived from biomarkers and chronological age and can be interpreted as the age at which the average mortality risk in NHANES III matches the predicted mortality risk (Levine, 2013; Levine et al., 2018). The computation procedure of BA involved two primary steps: Step 1 entailed training the algorithms in NHANES III and projecting biological aging measurements onto NHANES IV. In Step 2, these biological aging measurements were projected onto the UK Biobank dataset. The included biomarkers and their corresponding UK Biobank data fields are listed in Table S1, with BA values calculated as detailed in Text S1, Table S2, and Figure S2. The calculation of biological age values was performed using the R package “BioAge” (https://github.com/dayoonkwon/BioAge) (Kwon & Belsky, 2021).

The KDM‐BA prediction for an individual corresponds to the chronological age at which their physiology would be approximately normal. The KDM‐BA was derived through a series of regression analyses of individual biomarkers against chronological age within a reference population (Klemera & Doubal, 2006). The algorithm parameters are independently estimated for males and females. In our research, we utilized a set of nine biomarkers, specifically including forced expiratory volume in 1 s (FEV1), systolic blood pressure (SBP), albumin, alkaline phosphatase, blood urea nitrogen, creatinine, C‐reactive protein, glycated hemoglobin, and total cholesterol. The KDM‐BA formula is represented as follows:

$$\mathrm{KDM}-{\mathrm{BA}}_{\mathrm{EC}}=\frac{\sum_{i=1}^{9}\left(x_{i}-q_{i}\right)\frac{k_{i}}{s_{i}^{2}}+\frac{\mathrm{CA}}{s_{\mathrm{BA}}^{2}}}{\sum_{i=1}^{9}{\left(\frac{k_{i}}{s_{i}}\right)}^{2}+\frac{1}{s_{\mathrm{BA}}^{2}}}$$

In this calculation, *x* represents the value of biomarker i for an individual. CA is chronological age. For each biomarker *i*, the parameters *k*, *q*, and *s* are estimated from a regression of chronological age on the biomarker in the reference sample, where *k* is the intercept, *q* is the slope, and *s* is the root mean squared error. $s_{\mathrm{BA}}$ is a scaling factor equal to the square root of the variance in CA explained by the biomarker set in the reference sample.

The PhenoAge algorithm was derived from a multivariate analysis of mortality risk. The original PhenoAge algorithm was developed by an elastic‐net Gompertz regression of mortality for 42 biomarkers from NHANES III using 10‐fold cross‐validation to select the lambda parameter for penalized regression (Levine et al., 2018; Liu et al., 2018). To create a sparse and concise age estimator, prioritizing fewer biomarker variables for robust results, the lambda that minimized the mean squared error was chosen. This thorough analysis ultimately identified nine key clinical biomarkers: albumin, creatinine, glucose, C‐reactive protein, mean cell volume, lymphocyte proportion, red cell distribution width, alkaline phosphatase, white blood cell count, and chronological age. The PhenoAge formula is represented as follows:

$$\text{PhenoAge}=141.50225+\frac{\ln\left[-0.0055305\times \ln\left(1-\text{mortality risk}\right)\right]}{0.090165}$$

where

$$\text{mortality risk}=1-e^{-e^{\mathrm{xb}}\left[\exp\left(120\times \gamma \right)-1\right]/\gamma }$$

γ=0.007692696

$$\begin{array}{c} \mathrm{xb}=-19.90667-0.03359\times \text{albumin}+0.00951\times \text{creatinine}\\ \;+0.19532\times \text{glucose}+0.09537\times \ln\left(\;C-\text{reactive protein}\right)\\ \;-0.01200\times \text{lymphocyte percentage}+0.02676\times \text{mean cell volume}\\ \;+0.33062\times \mathrm{red}\;\text{cell distribution width}+0.00187\\ \;\times \text{alkaline phosphatase}+0.05542\times \text{white blood cell count}\\ \;+0.08035\times \text{chronological}\;\mathrm{age} \end{array}$$

We computed the BA values for each participant based on the above two algorithms (Mak et al., 2023). To quantify differences in BA and chronological age among participants, we calculated “age acceleration” values. These values represented the residuals obtained by regressing the KDM‐BA and PhenoAge values against chronological age, effectively measuring how much a participant's BA deviates from their chronological age (McEwen et al., 2018). A higher BA acceleration indicated that an individual is biologically older relative to their chronological age, whereas a lower BA acceleration suggested that an individual is biologically younger relative to their chronological age. To make effect sizes for the two measures comparable, BA acceleration values were standardized (mean = 0, SD = 1). The standardized BA acceleration values (KDM‐BA acceleration and PhenoAge acceleration) were then used to estimate their associations with stroke. Additionally, we categorized the BA acceleration values into four groups (low, medium‐low, medium‐high, and high) based on the quartile.

### 2.3 Assessment of polygenic risk score

To estimate genetic susceptibility, a PRS for stroke was constructed for each participant. In the UK Biobank, genotyping was conducted using the UK Biobank Axiom Array and the UK BiLEVE Axiom Array, followed by imputation using a combined reference panel of the Haplotype Reference Consortium and UK10K. Detailed information on genotyping, imputation, and quality control processes has been described previously (Bycroft et al., 2018). In this study, we constructed a weighted PRS for stroke using 87 single‐nucleotide polymorphisms (SNPs) significantly associated with stroke (*p* < 1 × 10^−5^) and with low linkage disequilibrium (*r* ^2^ < 0.05), identified from a GWAS conducted on an independent sample of individuals of European ancestry (selected SNPs are listed in Table S3) (Malik et al., 2018). The correlations between variants (*r* ^2^ < 0.05) were based on participants of European ancestry from the 1000 Genomes Project, phase 3, to avoid overestimation of the effect sizes of genetic variants. This approach has been validated in previous research (Kim et al., 2021; Rutten‐Jacobs et al., 2018), where the PRS calculated using these SNPs was shown to effectively predict stroke outcomes. The PRS was calculated using the following formula:

$$\mathrm{PRS}=\sum_{i=1}^{m}\beta_{i}\times {\mathrm{SNP}}_{i}$$

Where the SNP represents the number of risk alleles (recorded as 0, 1, 2) and the *β* coefficients were extracted from the reported GWAS data (Malik et al., 2018). A higher PRS indicates a higher genetic predisposition to stroke. The genetic risk was classified into low (bottom quintile), intermediate (quintiles 2–4), and high (top quintile) categories according to the distribution of PRSs.

### 2.4 Assessment of healthy behaviors score

The healthy behaviors score was based on the latest cardiovascular health indicators from the AHA, encompassing five behaviors: diet, physical activity, tobacco/nicotine exposure, sleep, and body mass index (BMI). The diet assessment included ten components: fruits, vegetables, whole grains, (shell) fish, dairy, vegetable oils, refined grains, processed meats, unprocessed meats, and sugar‐sweetened beverages (Table S4). Physical activity was measured by weekly minutes of moderate and vigorous activity. Nicotine exposure was determined by smoking status (never, former, current) and secondhand smoke exposure (yes/no). Former smokers were further categorized by quit duration (<1 year, 1–<5 years, ≥5 years). Sleep was assessed by asking, “How many hours of sleep do you get in 24 hours?” Each health behavior metric was scored from 0 to 100 points (Lloyd‐Jones et al., 2022). Detailed definitions, measurement, and scoring of health behavior metrics are presented in Table S5. The total health behaviors score for each individual was calculated by adding the scores for each of the five metrics and dividing the total score by five to create a health behaviors score ranging from 0 to 100 points. We categorized health behaviors score into low, moderate, and high levels by tertiles.

### 2.5 Assessment of covariates

We considered a series of demographic characteristics, socioeconomic status, and behavioral factors as covariates, including age, sex, assessment center, household income, years of education, employment status, Indices of Multiple Deprivation (IMD), and alcohol consumption. Household income was categorized as level 1 (< £18,000), level 2 (£18,000–30,999), level 3 (£31,000–51,999), level 4 (> £52,000). Years of education were categorized as 10 years or fewer, 11–15 years, or more than 15 years. Employment status was categorized into employed (including those in paid employment or self‐employment, doing unpaid or voluntary work, and full‐ or part‐time students) and unemployed. The IMD, which measures multiple deprivation at the small area level, was divided by quartile as categorical variables. Alcohol consumption was categorized as never, occasionally, 1–2 times per week, 3–4 times per week, and daily. We coded missing covariates as a missing indicator category for categorical variables and with mean values for continuous variables.

### 2.6 Assessment of stroke and its subtypes

The diagnoses of stroke were ascertained using hospital inpatient records (Hospital Episode Statistics for England, Morbidity Records for Scotland, and the Patient Episode Database for Wales) and death register data (National Health Service [NHS] Digital, NHS Central Register, and National Records) as per the algorithmically defined stroke outcomes listed in category 43. The outcome variables were the incidence of overall stroke as well as stroke subtypes, including ischemic stroke (IS), intracerebral hemorrhage (ICH), and subarachnoid hemorrhage (SAH). The incidence of each stroke type was determined based on the first occurrence of that type. Participants with stroke diagnosed or self‐reported before baseline were excluded from the analysis. Detailed codes from the International Classification of Disease (ICD)‐10, ICD‐9, and self‐reported used to identify participants with stroke and its subtypes are provided in Table S6. At the time of our analysis, the censoring dates for the Hospital Episode Statistics for England, Scottish Morbidity Records for Scotland, and Patient Episode Database for Wales were October 31, 2022; August 31, 2022; and May 31, 2022, respectively. The follow‐up period was determined from baseline to the earliest occurrence of stroke diagnosis, death, loss to follow‐up, or censoring.

### 2.7 Statistical analysis

The baseline characteristics of included participants were described using mean (standard deviation) for continuous variables and number (proportion) for categorical variables. Cox proportional hazard regression models were used to estimate the associations between BA acceleration with incident stroke and its subtypes to calculate the hazard ratio (HR) and 95% confidence interval (CI). The proportional hazards assumptions were verified by computing scaled Schoenfeld residuals and scrutinizing time‐based log HR plots. Model 1 was adjusted for age and sex. Model 2 was further adjusted for assessment center, household income, years of education, employment status, IMD, alcohol consumption, and behaviors score. Restricted cubic spline models with three knots (10th, 50th, and 90th) were performed to explore the dose–response associations between BA acceleration and the risk of incident stroke. Furthermore, we performed subgroup analyses to test whether the associations may differ by age, sex, household income, years of education, employment status, IMD, and alcohol consumption.

The joint analysis steps were as follows. We evaluated the joint associations between BA acceleration and genetic categories with incident stroke risk (12 categories with low genetic risk and low BA acceleration as reference), further adjusting for the genotype batch and the first ten genetic principal components. Moreover, we utilized the relative excess risk due to interaction (RERI) and the attributable proportion (AP) due to interaction to evaluate the additive interaction of genetic risk with two BA accelerations on the risk of stroke.

Moreover, mediation analysis was conducted using the R package “mediation” to evaluate whether BA acceleration mediated the relationship between behaviors score and stroke risk. This involved two models: a multivariate linear regression to assess the effect of behaviors score (exposure) on BA acceleration (mediator) and a multivariate survival regression to examine the impact of both behaviors score (exposure) and BA acceleration (mediator) on stroke risk (outcome). The mediation analysis provided estimates for the indirect effects (IE), the direct effect (DE), and the total effect (TE). The mediation proportion was derived by dividing IE by TE. To determine the 95% confidence intervals for the mediation proportion, a quasi‐Bayesian Monte Carlo simulation approach with 1000 iterations was employed.

To test the robustness of our results, we conducted a series of sensitivity analyses. First, we excluded the incident stroke events within two years before follow‐up to mitigate potential reverse causality. Second, we performed stratified analyses by age and follow‐up time using a time‐varying model with interaction terms between BA acceleration and age (in 5‐year intervals) or between BA acceleration and follow‐up time (in 3‐year intervals) to calculate HRs for different time and age periods. Third, considering the relatively high proportion of individuals with incomplete biological aging measurement information excluded from the primary analysis, when there was only one missing value among the nine biological markers in each algorithm, we imputed the missing value with the median to assess the imputed biological aging concerning stroke risk. Fourth, we conducted analyses using a dataset with no missing values for covariates to compare with the results based on imputation. Fifth, to mitigate potential bias stemming from competing events, we utilized the Fine and Gray subdistribution hazards regression models. In this approach, stroke was treated as the primary interest event, while death from other causes was regarded as a competing event.

All the above analyses were carried out using R version 4.2.3. A two‐sided *P* value of 0.05 or less was considered to indicate statistical significance.

## 3 RESULTS

### 3.1 Participant characteristics and biological age

In this study, a total of 253,932 participants with complete data were included. Baseline characteristics of the study population are presented in Table 1. During a total of 3,367,446 person‐years (median follow‐up 13.6 years), 5460 incident stroke cases were reported, of which 4337 were IS, 951 ICH, and 553 SAH. Compared to participants without stroke, those with stroke were older (median: 60.98 vs. 56.10 years), more likely to be male (55.6% vs. 44.6%), had less education (less than 10 years: 39.1% vs. 31.4%), lower income (less than £18,000: 26.1% vs. 17.0%), more often unemployed (54.3% vs. 36.1%), and had higher IMD (most deprived group: 23.8% vs. 20.9%) (Table 1). Participants who developed stroke were more likely to have less healthy behaviors score, higher genetic risk, older BA, and higher BA acceleration (Table 1).

**TABLE 1:** Baseline characteristics of study participants by incident stroke.

Characteristic	Non‐stroke (*n* = 248,472)	Incident stroke (*n* = 5460)	*p*‐Value
Age at baseline (years)	56.10 (8.03)	60.98 (6.75)	<0.001
Sex, *n* (%)			
Female	137,612 (55.4)	2422 (44.4)	<0.001
Male	110,860 (44.6)	3038 (55.6)	
Assessment center, *n* (%)			
England	226,905 (91.3)	4957 (90.8)	0.207
Scotland	10,322 (4.2)	253 (4.6)	
Wales	11,245 (4.5)	250 (4.6)	
Education, *n* (%)			
≤10	78,105 (31.4)	2135 (39.1)	<0.001
11–14	44,940 (18.1)	965 (17.7)	
>15	123,906 (49.9)	2308 (42.3)	
Unknown	1521 (0.6)	52 (1.0)	
Household income, £, *n* (%)			
<18,000	42,137 (17.0)	1426 (26.1)	<0.001
18,000–30,999	54,443 (21.9)	1372 (25.1)	
31,000–51,999	59,874 (24.1)	1054 (19.3)	
>52,000	62,256 (25.1)	800 (14.7)	
Unknown	29,762 (12.0)	808 (14.8)	
Employment status, *n* (%)			
Employed	158,075 (63.6)	2484 (45.5)	<0.001
Unemployed	89,821 (36.1)	2964 (54.3)	
Unknown	576 (0.2)	12 (0.2)	
Indices of Multiple Deprivation, *n* (%)			
Q1 (Least deprived)	66,449 (26.7)	1346 (24.7)	<0.001
Q2	64,004 (25.8)	1346 (24.7)	
Q3	59,667 (24.0)	1319 (24.2)	
Q4 (Most deprived)	52,006 (20.9)	1298 (23.8)	
Unknown	6346 (2.6)	151 (2.8)	
Alcohol intake, *n* (%)			
Never	14,668 (5.9)	460 (8.4)	<0.001
Occasionally	52,503 (21.1)	1160 (21.2)	
1–2 times a week	65,679 (26.4)	1274 (23.3)	
3–4 times a week	61,780 (24.9)	1183 (21.7)	
Daily or almost daily	53,756 (21.6)	1380 (25.3)	
Unknown	86 (0.0)	3 (0.1)	
Total behaviors score	67.34 (13.65)	65.25 (14.15)	<0.001
Polygenic risk score	5.45 (0.55)	5.50 (0.55)	<0.001
Biological ages			
KDM‐BA	53.70 (9.14)	59.60 (8.09)	<0.001
KDM‐BA acceleration	−0.02 (4.36)	0.99 (4.69)	<0.001
PhenoAge	49.27 (9.28)	55.49 (8.45)	<0.001
PhenoAge acceleration	−0.03 (4.47)	1.25 (5.19)	<0.001
Components of biological ages			
FEV1 (L)	2.91 (0.79)	2.75 (0.78)	<0.001
SBP (mm Hg)	137.46 (18.40)	145.60 (19.60)	<0.001
Total Cholesterol (mg/dL)	223.72 (42.71)	222.25 (44.70)	0.012
Glycated hemoglobin (%)	5.40 (0.53)	5.56 (0.71)	<0.001
Albumin (g/dL)	4.53 (0.26)	4.48 (0.26)	<0.001
Creatinine (mg/dL)	0.81 (0.17)	0.84 (0.19)	<0.001
C‐reactive protein (mg/dL)	0.24 (0.40)	0.30 (0.46)	<0.001
Alkaline phosphatase (U/L)	82.45 (25.42)	87.11 (25.85)	<0.001
Blood urea nitrogen (mg/dL)	15.06 (3.65)	15.89 (4.25)	<0.001
Lymphocyte (%)	28.90 (7.24)	28.10 (7.64)	<0.001
Mean cell volume (fL)	82.79 (5.20)	83.40 (5.53)	<0.001
Serum glucose (mmol/L)	5.07 (1.09)	5.29 (1.55)	<0.001
Red cell distribution width (%)	13.43 (0.91)	13.55 (0.96)	<0.001
White blood cell count (1000 cells/μL)	6.80 (1.79)	7.12 (2.09)	<0.001

*Note*: Mean values (standard deviation) for continuous variables and *n* (%) for categorical variables. *p* values are derived using either Student's *t* test or Chi‐square test.

Abbreviations: FEV1, forced expiratory volume in 1‐second; KDM‐BA, Klemera‐Doubal Method Biological Age; PhenoAge, Phenotypic Age; SBP, systolic blood pressure.

Correlations between the BA measures and chronological age in the UK Biobank are shown in Figure S2. Chronological age was highly correlated with both KDM‐BA (*r* = 0.88) and PhenoAge (*r* = 0.88), and KDM‐BA was highly correlated with PhenoAge (*r* = 0.84) in the UK Biobank. However, KDM‐BA acceleration was weakly correlated with the PhenoAge acceleration (*r* = 0.29).

### 3.2 Associations of biological age accelerations with the incidence of stroke

Table 2 presents the relations of two BA acceleration values with the risk of stroke and stroke subtypes. After adjusting for potential confounders, both BA accelerations were associated with an elevated risk of stroke (KDM‐BA acceleration: HR per 1‐SD increase = 1.28, 95% CI = 1.25–1.32; PhenoAge acceleration: 1.22, 1.19–1.25). In categorical variable (quartiles) analysis, the risks of stroke increased significantly across two BA acceleration categories (all *p* for trend <0.001). Compared to low KDM‐BA acceleration, the HRs of stroke for the highest groups were 1.76 (95% CI: 1.63–1.91). Likewise, the risk of stroke was increased in the highest group compared with low PhenoAge acceleration (HR: 1.54, 95% CI: 1.42–1.67). For the stroke subtypes, two BA accelerations were associated with an elevated risk of IS, ICH, and SAH subtypes (KDM‐BA acceleration for IS: 1.32, 1.28–1.36; PhenoAge acceleration for IS: 1.26, 1.22–1.29; KDM‐BA acceleration for ICH: 1.15, 1.08–1.23; PhenoAge acceleration for ICH: 1.08, 1.02–1.16; KDM‐BA acceleration for SAH: 1.16, 1.07–1.27; PhenoAge acceleration for SAH: 1.08, 1.00–1.18). Similar associations were also observed when two BA accelerations were analyzed as categorical variables (quartiles), with *p* for trend <0.05.

**TABLE 2:** Association between biological age accelerations and risk of stroke and stroke subtypes.

Characteristic	Cases/participants	Person‐years	Model 1		Model 2	
			HR (95% CI)	*p*‐Value	HR (95% CI)	*p*‐Value
Stroke						
KDM‐BA acceleration						
Quartile						
Q1	1103/63483	846,597	1 (Reference)		1 (Reference)	
Q2	1204/63483	845,298	1.21 (1.12–1.32)	<0.001	1.17 (1.07–1.27)	<0.001
Q3	1339/63483	843,369	1.40 (1.29–1.52)	<0.001	1.30 (1.20–1.41)	<0.001
Q4	1814/63483	832,182	1.99 (1.84–2.15)	<0.001	1.76 (1.63–1.91)	<0.001
*p* for trend				<0.001		<0.001
Per SD increment			1.34 (1.30–1.37)	<0.001	1.28 (1.25–1.32)	<0.001
PhenoAge acceleration						
Quartile						
Q1	1063/63483	854,349	1 (Reference)		1 (Reference)	
Q2	1082/63483	849,453	0.99 (0.91–1.08)	0.77	0.96 (0.88–1.04)	0.328
Q3	1374/63483	843,251	1.23 (1.13–1.33)	<0.001	1.16 (1.07–1.25)	<0.001
Q4	1941/63483	820,393	1.73 (1.61–1.87)	<0.001	1.54 (1.42–1.67)	<0.001
*p* for trend				<0.001		<0.001
Per SD increment			1.27 (1.24–1.30)	<0.001	1.22 (1.19–1.25)	<0.001
Ischemic stroke						
KDM‐BA acceleration						
Quartile						
Q1	845/63483	847,401	1 (Reference)		1 (Reference)	
Q2	957/63483	846,260	1.28 (1.17–1.41)	<0.001	1.23 (1.12–1.35)	<0.001
Q3	1065/63483	844,373	1.50 (1.37–1.64)	<0.001	1.38 (1.26–1.51)	<0.001
Q4	1470/63483	833,472	2.19 (2.00–2.38)	<0.001	1.91 (1.75–2.09)	<0.001
*p* for trend				<0.001		<0.001
Per SD increment			1.38 (1.34–1.42)	<0.001	1.32 (1.28–1.36)	<0.001
PhenoAge acceleration						
Quartile						
Q1	782/63483	855,379	1 (Reference)		1 (Reference)	
Q2	844/63483	850,327	1.03 (0.94–1.14)	0.537	1.00 (0.90–1.10)	0.961
Q3	1107/63483	844,098	1.31 (1.19–1.44)	<0.001	1.23 (1.12–1.35)	<0.001
Q4	1604/63483	821,701	1.89 (1.73–2.06)	<0.001	1.66 (1.51–1.81)	<0.001
*p* for trend				<0.001		<0.001
Per SD increment			1.31 (1.28–1.35)	<0.001	1.26 (1.22–1.29)	<0.001
Intracerebral hemorrhage						
KDM‐BA acceleration						
Quartile						
Q1	230/63483	850,148	1 (Reference)		1 (Reference)	
Q2	205/63483	849,469	0.96 (0.79–1.16)	0.647	0.93 (0.77–1.13)	0.475
Q3	226/63483	848,244	1.07 (0.89–1.29)	0.457	1.02 (0.85–1.24)	0.799
Q4	290/63483	838,627	1.42 (1.19–1.70)	<0.001	1.31 (1.09–1.58)	0.003
*p* for trend				<0.001		0.002
Per SD increment			1.19 (1.11–1.26)	<0.001	1.15 (1.08–1.23)	<0.001
PhenoAge acceleration						
Quartile						
Q1	227/63483	857,895	1 (Reference)		1 (Reference)	
Q2	194/63483	853,038	0.85 (0.70–1.03)	0.092	0.83 (0.68–1.01)	0.058
Q3	238/63483	848,278	1.02 (0.85–1.23)	0.809	0.98 (0.82–1.18)	0.852
Q4	292/63483	827,277	1.26 (1.05–1.50)	0.011	1.16 (0.96–1.39)	0.121
*p* for trend				0.002		0.033
Per SD increment			1.12 (1.05–1.19)	<0.001	1.08 (1.02–1.16)	0.014
Subarachnoid hemorrhage						
KDM‐BA acceleration						
Quartile						
Q1	113/63483	850,358	1 (Reference)		1 (Reference)	
Q2	129/63483	849,576	1.10 (0.85–1.41)	0.474	1.08 (0.83–1.39)	0.571
Q3	125/63483	848,276	1.04 (0.80–1.34)	0.780	1.00 (0.77–1.30)	0.995
Q4	186/63483	838,688	1.53 (1.21–1.95)	<0.001	1.43 (1.12–1.82)	0.005
*p* for trend				0.001		0.006
Per SD increment			1.20 (1.10–1.30)	<0.001	1.16 (1.07–1.27)	0.001
PhenoAge acceleration						
Quartile						
Q1	133/63483	858,025	1 (Reference)		1 (Reference)	
Q2	120/63483	853,084	0.97 (0.76–1.24)	0.799	0.95 (0.74–1.22)	0.696
Q3	141/63483	848,361	1.18 (0.93–1.50)	0.180	1.14 (0.89–1.45)	0.298
Q4	159/63483	827,429	1.39 (1.10–1.75)	0.006	1.28 (1.01–1.63)	0.043
*p* for trend				0.002		0.018
Per SD increment			1.12 (1.03–1.21)	0.006	1.08 (1.00–1.18)	0.057

*Note*: Model 1: Adjusted for age and sex. Model 2: Further adjusted for assessment center, household income, years of education, employment status, IMD, alcohol consumption, and behaviors score.

Abbreviations: CI, confidence interval; HR, hazard ratio; KDM‐BA, Klemera‐Doubal Method Biological Age; PhenoAge, Phenotypic Age; Q, Quartile; SD, standard deviation.

**FIGURE 1:**
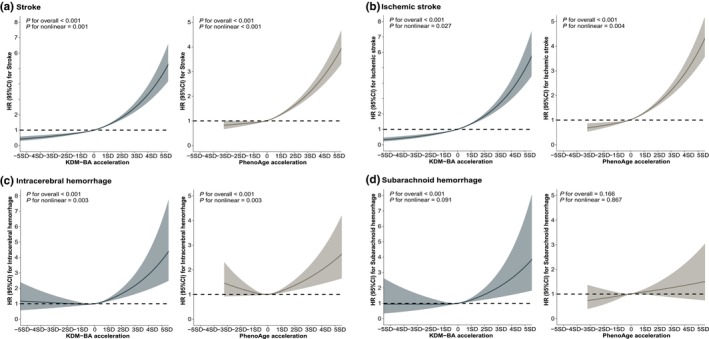
Association between biological age accelerations with the risk of stroke and stroke subtypes using restricted cubic splines models with three knots. Models adjusted for age, sex, assessment center, household income, years of education, employment status, IMD, alcohol consumption, and behaviors score.

In dose–response analysis, restricted cubic spline curves indicated the nonlinear positive associations between KDM‐BA and PhenoAge acceleration with overall stroke, IS, and ICH subtypes (*p* for overall <0.05, *p* for nonlinearity <0.05) (Figure 1). For overall stroke and IS, the dose–response curves for KDM‐BA and PhenoAge acceleration showed a significant protective effect when BA acceleration was below zero, while the risk of stroke sharply increased when BA acceleration exceeded zero (Figure 1a,b). For ICH, a significant risk effect was observed only when KDM‐BA and PhenoAge acceleration were above zero, with a similar pattern seen in KDM‐BA acceleration for SAH (Figure 1c,d).

**FIGURE 2:**
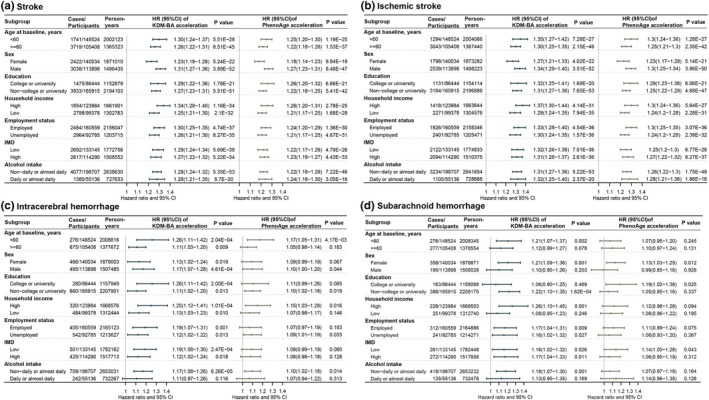
Subgroup analysis by population characteristics for the association between biological age accelerations with the risk of stroke and stroke subtypes. Models adjusted for age, sex, assessment center, household income, years of education, employment status, Index of Multiple Deprivation, alcohol consumption, and behaviors score.

Stratified analyses revealed that BA acceleration significantly increased the risk of stroke and IS across all subgroups. For ICH and SAH, there was also a trend toward increased risk, although some subgroups did not reach statistical significance, possibly due to limited sample sizes (Figure 2). Overall, the effect of BA acceleration on the risk of stroke and its subtypes was more pronounced among participants who were under 60 years old, had higher education (university level or above), had higher income, were employed, or had low IMD, reflecting a broad trend across most subgroups (Figure 2).

### 3.3 Joint effects of biological age accelerations and genetic susceptibility on stroke risk

Single analyses of PRS showed that individuals with higher PRS levels were more likely to develop overall stroke, IS, and ICH subtypes, but not SAH, during the follow‐up period (Table S7). We also observed a significant linear positive association between PRS and the risk of overall stroke, IS, and ICH subtypes, but no significant association with SAH (*p* for overall >0.05, *p* for nonlinear >0.05, Figure S3).

**FIGURE 3:**
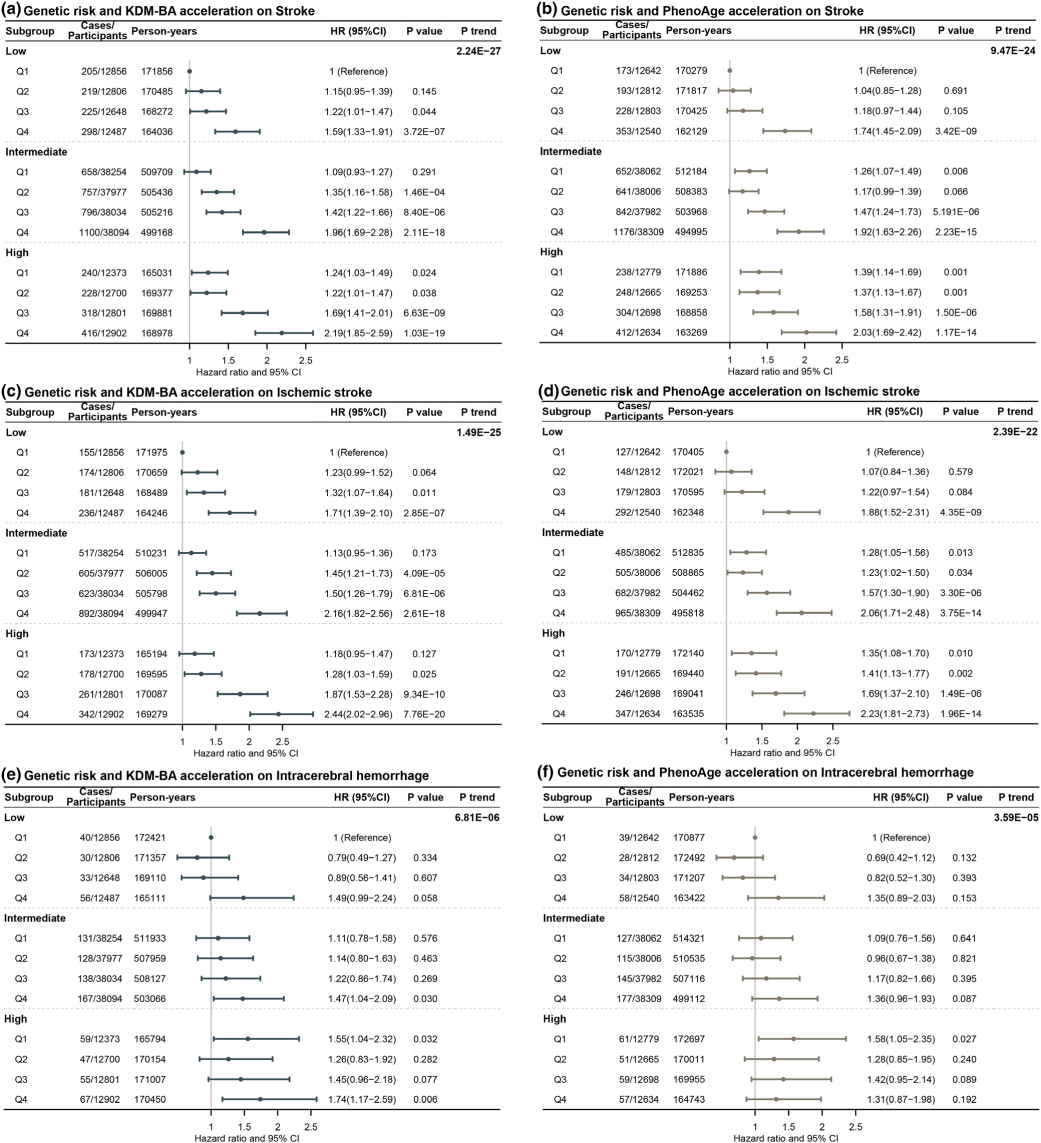
The joint associations of biological age accelerations and genetic risk score with the risk of stroke and stroke subtypes. Models adjusted for age, sex, assessment center, household income, years of education, employment status, Index of Multiple Deprivation, alcohol consumption, behaviors score, genotype batch, and the first ten genetic principal components.

The joint associations of genetic susceptibility and BA accelerations are reported in Figure 3. The risk of overall stroke increased with genetic risk and BA acceleration categories, and this trend is also significant for IH and ICH subtypes (*p* for trend <0.001). Moreover, compared to participants with the lowest PRS and the lowest KDM‐BA acceleration, those with the highest PRS and the highest BA acceleration had the highest stroke risk (high PRS and Q4 of KDM‐BA acceleration: 2.19, 1.85–2.59; high PRS and Q4 of PhenoAge acceleration: 2.03, 1.69–2.42). The additive interactions of PRS and BA acceleration are shown in Table S8. The RERI and AP values were 0.35 (0.02–0.68) and 0.16 (0.01–0.31) for the highest PRS and the highest KDM‐BA acceleration groups. These findings suggested that the interaction contributed to a 0.35 relative excess risk and explained 16% of the stroke risk.

### 3.4 Mediation effects of biological age accelerations

We observed a higher healthy behaviors score was associated with a decreased risk of overall stroke, IS, and ICH subtypes, but not SAH (Table S9). There was a significant linear negative association between healthy behaviors score and overall stroke, IS and ICH subtypes risk (*p* for overall <0.05, *p* for nonlinear >0.05, Figure S4), but no significant association with SAH (*p* for overall >0.05, *p* for nonlinear >0.05, Figure S4).

**FIGURE 4:**
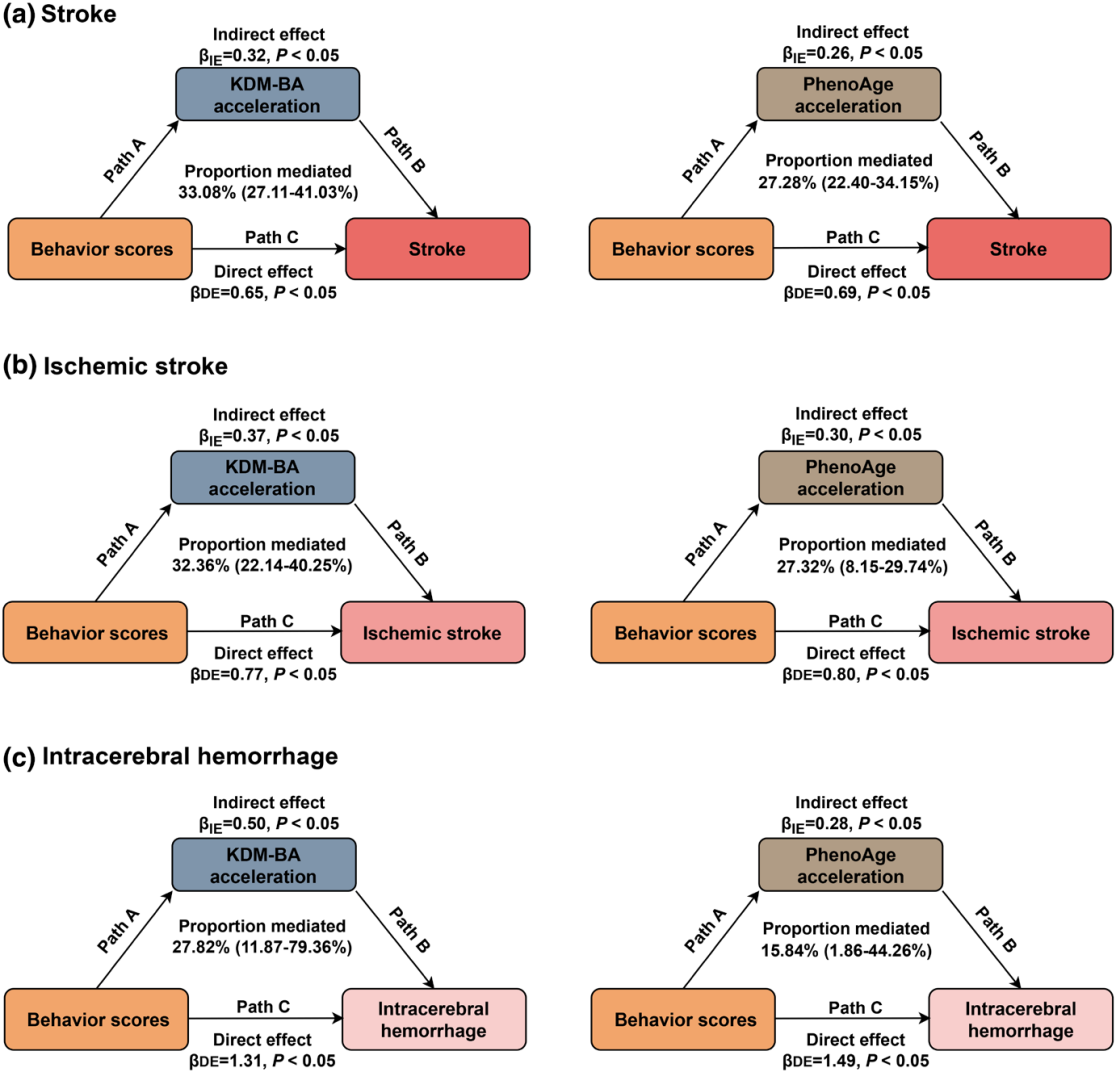
Mediation analysis of behaviors score on the association between biological age accelerations and risk of stroke and stroke subtypes. Indirect effects, direct effect and proportion mediated were computed by the casual mediation analysis. 95% confidence intervals were generated from 1000 bootstrap samples. Covariates included age, sex, assessment center, years of education, household income, employment status, Index of Multiple Deprivation, and alcohol consumption.

Furthermore, the mediation proportion of KDM‐BA acceleration in associations of behaviors score with risk of overall stroke, IS, and ICH was 33.08% (27.11%–41.03%), 32.36% (22.14%–40.25%), and 27.82% (11.87%–79.36%), respectively. Similarly, the associations between behaviors score with overall stroke, IS, and ICH mediated by PhenoAge acceleration, with a mediation proportion of 27.28% (22.40%–34.15%) for overall stroke, 27.32% (8.15%–29.74%) for IS, and 15.84% (1.86%–44.26%) for ICH, respectively (Figure 4).

### 3.5 Sensitivity analyses

We conducted several further sensitivity analyses. First, we repeated the analysis restricting the sample to participants with >2 years of follow‐up and found both measures of BA acceleration showed robust associations with incident stroke (Table S10). Second, although the proportional hazards assumption was considered reasonable, as evidenced by the approximately parallel log (HR) curves for KDM‐BA and PhenoAge acceleration over follow‐up time (Figure S5), we conducted stratified time‐varying models. The results indicated that the relationship between KDM‐BA and PhenoAge acceleration and stroke risk remained consistent across different age groups and follow‐up intervals, without any abnormal variations (Figures S6–S9). Third, the associations were also consistent when analyzing biomarkers with only one missing value imputed using the median or conducting analyses excluding missing covariate values (Tables S11 and S12). Finally, considering deaths from other causes as competing events, the results from the competing risks model were consistent with the primary analyses regarding the association between BA acceleration and stroke risk (Table S13).

## 4 DISCUSSION

Our study evaluated the associations between BA acceleration and the incidence of overall stroke and its subtypes among 253,932 midlife and older adults in the UK Biobank. The main finding was that adults with more advanced BA had a higher risk of incident overall stroke and IS, ICH, and SAH subtypes at 13.6 years of follow‐up compared with peers who have the same chronological age but younger BA. The risks of overall stroke and IS and ICH subtypes increased with genetic risk and BA acceleration categories. We also found significant additive interactions of high genetic risk with high KDM‐BA acceleration on the risk of incident stroke. Furthermore, our results indicated that the protective effect of healthy behaviors score on stroke and its subtypes was partially mediated by a reduction in BA acceleration.

There is increased evidence of an association between biological aging and stroke; however, the accurate measurement of BA is the key to exploring the association. Previous studies have focused on the association between several BA measures and stroke, including telomere length (D'Mello et al., 2015; Waziry et al., 2023; Zhang et al., 2023), epigenetic age calculated based on DNA methylation (Soriano‐Tárraga et al., 2016), and retinal age calculated from fundus images (Zhu et al., 2022). However, these measures are typically based on omics data with limited sample sizes (usually ranging from hundreds to thousands), and standardizing their detection in practical applications is also challenging. Previous studies found that individual clinical biomarkers covering a range of organs and systems (e.g., cardiometabolic (Au Yeung et al., 2018; Kim et al., 2018; Lee et al., 2018), inflammatory (Low et al., 2019), and organ functions (Kühn et al., 2021; Silvestre et al., 2018)) have different effects on stroke and its subtypes. In this study, we introduced the methods of the KDM‐BA and the PhenoAge algorithm, which integrate single routine clinical or blood biomarkers to evaluate BA. The clinical indicators common to both methods include C‐reactive protein, albumin, alkaline phosphatase, and creatinine, which reflect the status of systemic inflammation, liver function, and kidney function. However, the specific indicators of KDM‐BA focus on clinical biomarkers related to lung function (FEV1), blood pressure (SBP), lipid metabolism (total cholesterol), and glucose metabolism (glycated hemoglobin), which are closely associated with cardiovascular system metabolism. In contrast, the specific indicators of PhenoAge place greater emphasis on assessing inflammation and immune status, such as white blood cell count, lymphocyte proportion, mean cell volume, and red cell distribution width. These findings align with previous GWAS studies showing that KDM‐BA primarily captures cardiovascular and metabolic aging, while PhenoAge reflects inflammation/immune aging (Kuo et al., 2021). Despite capturing different aging dimensions, both BA measures indicate that accelerated biological aging increases the risk of stroke and its subtypes. These finding further underscores the importance of BA acceleration as an independent risk factor for stroke, offering new insights for future prevention and intervention strategies.

To more comprehensively understand the association between accelerated biological aging and the occurrence of stroke, it is necessary to explore various possible biological mechanisms. First, metabolic abnormalities are a key factor. Chronic hypertension can lead to thickening and stiffening of the vessel walls (Boutouyrie et al., 2021). Dysregulation of glucose metabolism, resulting in hyperglycemia and insulin resistance, can increase oxidative stress and inflammation in endothelial cells (Yan et al., 2003). Lipid metabolism disorders contribute to the deposition of lipids in the vessel walls. These metabolic issues collectively impair vascular health, reduce vascular elasticity, and accelerate vascular aging, promoting the development of atherosclerosis (Gepner et al., 2014). This increases the risk of vascular occlusion or rupture, thereby significantly elevating the risk of stroke. Second, chronic inflammation and immune responses play a crucial role in this process. As biological aging progresses, systemic inflammation levels gradually increase, often referred to as “inflammaging” (Ajoolabady et al., 2023; Li et al., 2023). This low‐grade chronic inflammatory state not only accelerates the aging and dysfunction of endothelial cells but also leads to increased permeability of the vessel walls, promoting the formation of atherosclerosis (Ajoolabady et al., 2023). Additionally, inflammation may activate abnormal immune cell activation within the immune system. The accumulation of these cells in damaged vessels and the secretion of inflammatory factors can further exacerbate vascular damage, thereby increasing the risk of stroke. Third, multi‐organ dysfunction is a significant manifestation of accelerated biological aging. Declines in liver and kidney function can impair the clearance of metabolic waste and lead to the accumulation of toxins in the body, while reduced lung function can affect oxygen supply. These dysfunctions increase the systemic metabolic burden, potentially disrupting blood circulation and exacerbating vascular damage, thereby increasing the risk of stroke.

To our knowledge, this is the first study to jointly consider the effects of accelerated biological aging and genetic risk on the risk of stroke. Our results indicate that, compared to the lowest group of both BA acceleration and PRS, the highest group of both BA acceleration and PRS is associated with approximately a two‐fold increase in the risk of stroke. Additionally, we found a potential additive interaction between KDM‐BA acceleration and PRS of stroke. Specifically, high KDM‐BA acceleration may amplify the genetic risk of stroke. This is biologically plausible for several reasons. First, previous GWAS findings have shown that KDM‐BA acceleration emphasizes capturing cardiovascular metabolic aging processes (Kuo et al., 2021). Therefore, from a genetic perspective, KDM‐BA acceleration is more likely to synergize with stroke PRS. Second, some SNPs in the stroke PRS map to genes such as CDKN1A, CHEK2, CDK6, and SMARCE1, which have been shown to be closely associated with DNA damage, cellular aging, and the cell cycle, reflecting accelerated biological aging. From a public health perspective, the observed interaction between accelerated biological aging and stroke genetic factors helps identify high‐risk subgroups, which could potentially guide primary prevention strategies for stroke.

Notably, given that genetic background is generally difficult to alter, it becomes increasingly urgent to address ways to slow accelerated biological aging. Evidence from the Health and Retirement Study suggests that 29.2% of the variance in phenotypic age can be attributed to 11 research domains, with behavior contributing the most to phenotypic age (9.2%) (Liu et al., 2019). Li et al. found that BA acceleration mediated approximately 20% of the association between lifestyle and the risk of overall cardiovascular disease (Li et al., 2024). Our study incorporated five healthy behaviors based on the latest AHA's criteria and calculated behaviors score based on detailed scoring guidelines. Five behaviors, including eating better, being more active, quitting tobacco, getting healthy sleep, and managing weight, could alter the aging process (Gao et al., 2022; Lin, 2022). Our study found that in the process of reducing stroke and its subtypes risk through healthy behaviors, approximately 15.84%–33.08% of the mediation effect was mediated by the reduction in BA acceleration. These results suggest that establishing targeted prevention strategies on modifiable behaviors is crucial for effectively mitigating BA acceleration and thereby reducing the risk of stroke.

In addition, we also explored the association between BA acceleration and stroke subtypes. The results indicate a significant positive correlation between BA acceleration with IS, ICH, and SAH, though the pathogenic mechanisms differ slightly. IS is due to basilar artery stenosis, cerebral vasospasm, and thrombosis caused by cerebral insufficiency. Accelerated biological aging can lead to endothelial cell damage and lipid deposition in the arterial walls, forming atherosclerotic plaques. These plaques narrow the vessel lumen, restrict blood flow, and increase the risk of ischemic stroke (Chen et al., 2023). Hemorrhagic stroke encompasses ICH and SAH. ICH, a non‐traumatic intracerebral hemorrhage, is associated with hypertension, diabetes, and other atherosclerosis risk factors. As BA increases, blood vessel elasticity decreases, and arterial walls gradually harden. This arterial stiffening makes blood vessels more prone to rupture under the pressure of hypertension, leading to hemorrhagic stroke. Aging also exacerbates microvascular disease, further increasing the risk of amyloid angiopathy, which makes blood vessels more fragile and raises the likelihood of hemorrhage. SAH is the rupture of blood vessels between the arachnoid membrane and the surface of the brain, caused by aneurysm or trauma. Accelerated biological aging may cause abnormal cerebrovascular structures, such as reduced vessel wall elasticity and poor vascular remodeling. These structural abnormalities heighten the risk of aneurysms and vascular malformations, leading to subarachnoid hemorrhage (Magid‐Bernstein et al., 2022).

Strengths of the study include the large sample size and relatively long follow‐up period, which allowed us to evaluate the impact of accelerated biological aging based on multiple clinical aging biomarkers on stroke and its subtypes. There are also some limitations in the study. First, our analysis was based solely on baseline clinical biomarkers. Although we used time‐varying models in the sensitivity analysis, we were unable to assess how changes in BA over time might influence stroke risk. Second, when measuring biological aging using biomarkers, our algorithm did not account for the effects of antihypertensive, cholesterol‐lowering, and antidiabetic medications. Future research should consider adjusting for these medications to enhance the accuracy and clinical relevance of biological aging assessments. Third, currently, independent PRSs for stroke subtypes have not been developed, and future research should focus on exploring genetic risk prediction for these subtypes, especially for subarachnoid hemorrhage, once larger sample sizes are available. Fourth, the UK Biobank sample comprised predominantly white participants, which may limit the generalizability of our results to other populations. Finally, as in other observational studies, although we carefully adjusted for stroke risk factors, the possibility of residual and unmeasured confounding cannot be ruled out.

## 5 CONCLUSIONS

In conclusion, this study reveals a significant association between accelerated biological aging and the risk of stroke and its subtypes. When accelerated biological aging coexists with a high genetic risk for stroke, individuals face the highest risk, and accelerated biological aging may amplify the effect of genetic factors on stroke development. Additionally, eating better, being more active, quitting tobacco, getting healthy sleep, and managing weight can effectively slow biological aging and reduce stroke risk. These findings highlight the clinical value of accelerated biological aging as a novel risk indicator, guiding risk stratification for high‐risk stroke populations and supporting health management strategies to delay biological aging to provide a scientific basis for stroke prevention.

## AUTHOR CONTRIBUTIONS

M.L., X.Y., T.Z., and X.Z. contributed to the conception of the study. X.Z. performed the analyses and wrote the draft of the manuscript. H.Z., Z.L., X.L., and Y.Z. contributed to suggestions on statistical analysis and interpretation of findings. M.L., X.Y., and T.Z. critically revised the manuscript. All authors read and approved the final version of the manuscript.

## FUNDING INFORMATION

This study was supported by the Taishan Scholars Program of Shandong Province (tstp20230654, tsqn202312328), the Excellent Youth Innovation Team of Shandong Provincial Higher Education Institutions (2022KJ012), and the Shandong Provincial Natural Science Foundation (ZR2022QH162). The funders were not involved in the collection, analysis, or interpretation of data, or the writing or submitting of this report.

## CONFLICT OF INTEREST STATEMENT

None declared.

## Supporting information


Appendix S1.

## Data Availability

The data are available from the UK Biobank on request (www.ukbiobank.ac.uk/).
