# Supplementary material for: Accelerated biological aging, healthy behaviors, and genetic susceptibility with incidence of stroke and its subtypes: A prospective cohort study
Source: Aging Cell. 2024 Dec 4;24(4):e14427. doi: 10.1111/acel.14427 (PMC11984664; doi:10.1111/acel.14427)
Supplement: Supplementary file 1 — Appendix S1. [file ACEL-24-e14427-s001.pdf]

Supplementary Materials

Accelerated biological aging, healthy behaviors, and genetic susceptibility with incidence of stroke and its subtypes: A prospective cohort study.

Table of Content

Text S1. The computation procedure of biological age 3

Table S1. Biomarkers included in the biological age algorithm and their field IDs in UK Biobank. 5
Table S2. Associations of biological age measures with mortality in NHANES IV. 6
Table S3. Information of genetic variants associated with stroke in the UK Biobank. 7
Table S4. Components of Dietary Recommendations for Adherence to AHA Guidelines in the UK Biobank 10
Table S5. Methods for evaluating each individual healthy behavior in the UK Biobank. 12
Table S6. Detailed codes from ICD-10, ICD-9, and self-reported used to identify participants with stroke and its subtypes. 14
Table S7. Association between polygenic risk score and risk of stroke and stroke subtypes. 15
Table S8. Additive interactions between biological age accelerations and genetic score and the risk of stroke and stroke subtypes. 17
Table S9. Association between healthy behaviors score and risk of stroke and stroke subtypes. 19
Table S10. Association between biological age accelerations and risk of stroke and stroke subtypes after excluding participants diagnosed with stroke within the first two years of follow-up. 21
Table S11. Association between biological age accelerations and risk of stroke and stroke subtypes for nine biomarkers with only one missing value imputed using the median. 24
Table S12. Association between biological age accelerations and risk of stroke and stroke subtypes using a data set without missing covariates. 27
Table S13. Association between biological age accelerations and risk of stroke and stroke subtypes using competitive risk model. 30

Figure S1. Flow diagram for inclusion of participants in this study. 33
Figure S2. Correlations between chronological age, biological ages, and age accelerations in (a) NHANES IV and (b) UK Biobank. 34
Figure S3. Association between genetic risk score and risk of stroke and stroke subtypes using restricted cubic splines models with three knots. 35
Figure S4. Association between total health behaviors score and risk of stroke and stroke subtypes using restricted cubic splines models with three knots. 36
Figure S5. Scaling Schoenfeld residuals and time-based logarithmic HR plots of biological age acceleration for stroke and subtypes. 37
Figure S6. Association between biological age accelerations and risk of stroke using time-varying model with interaction terms between KDM-BA or PhenoAge acceleration and age (in 5-year

intervals) (a and c) or between KDM-BA or PhenoAge acceleration and follow-up time (in 3-year intervals) (b and d). 38

Figure S7. Association between biological age accelerations and risk of Ischemic stroke (IS) using time-varying model with interaction terms between KDM-BA or PhenoAge acceleration and age (in 5-year intervals) (a and c) or between KDM-BA or PhenoAge acceleration and follow-up time (in 3-year intervals) (b and d). 39

Figure S8. Association between biological age accelerations and risk of Intracerebral hemorrhage (ICH) using time-varying model with interaction terms between KDM-BA or PhenoAge acceleration and age (in 5-year intervals) (a and c) or between KDM-BA or PhenoAge acceleration and follow-up time (in 3-year intervals) (b and d). 40

Figure S9. Association between biological age accelerations and risk of Subarachnoid hemorrhage (SAH) using time-varying model with interaction terms between KDM-BA or PhenoAge acceleration and age (in 5-year intervals) (a and c) or between KDM-BA or PhenoAge acceleration and follow-up time (in 3-year intervals) (b and d). 41

Text S1. The computation procedure of biological age

The computation procedure of biological age (BA) involves involved two primary steps:

Step 1 entailed training the algorithms in NHANES III and projecting biological aging measurements onto NHANES IV.

We identified 14 potential routinely collected biomarkers from various organ systems available in both NHANES III and UK Biobank. Specifically, the Klemera-Doubal Method Biological Age (KDM-BA) was calculated using forced expiratory volume in 1-second (FEV1), systolic blood pressure (SBP), and seven blood chemical parameters (albumin, alkaline phosphatase, blood urea nitrogen, creatinine, C-reactive protein, glycated hemoglobin, and total cholesterol), with separate estimations for males and females. Phenotypic Age (PhenoAge) was derived from nine blood chemistry measurements: albumin, creatinine, glucose, C-reactive protein, mean cell volume, lymphocyte proportion, red cell distribution width, alkaline phosphatase, white blood cell count, and chronological age. Of which, the clinical markers shared by both methods include albumin, creatinine, C-reactive protein, and alkaline phosphatase. These markers reflect systemic inflammation, kidney function, and liver function, all of which are crucial for assessing an individual's aging process. However, the specific markers of KDM-BA primarily focus on clinical biomarkers related to blood pressure (SBP), lipid metabolism (total cholesterol), glucose metabolism (glycated hemoglobin), and lung function (FEV1). These markers reflect the functional status of the metabolic, cardiovascular, and respiratory systems, and abnormalities in these systems are closely related to aging. In contrast, the specific markers of PhenoAge focus more on assessing inflammaging/immunosenescence, such as white blood cell count, lymphocyte proportion, mean cell volume, and red blood cell distribution width. These indicators reflect the activity

level of the immune system and the oxygen-carrying capacity of the blood, which are also key factors in the aging process. Based on previous research, we selected non-pregnant individuals aged 30-75 with complete biomarker data as the reference group for KDM-BA. For PhenoAge, our reference group included individuals aged 20-84 with complete biomarker data.

We then evaluated the performance of our BA algorithm in predicting mortality using a distinct NHANES IV cohort (recruited between 1999 and 2017). Our BA algorithms were statistically significantly associated with mortality during a median follow-up time of 7.4 years (quartile range: 4.1-11.5 years, Table S1). Both the KDM-BA and PhenoAge showed strong correlations with chronological age ($r > 0.95$), and Both of the BA metrics demonstrated a strong correlation in NHANES IV ($r = 0.96$, Figure S2A)

In Step 2, these biological aging measurements were projected onto the UK Biobank dataset.

In the final dataset from the UK Biobank, which included 253,932 participants with complete data on all 14 biomarkers, both the KDM-BA and PhenoAge showed strong correlations with chronological age ($r > 0.85$), and both of the BA metrics demonstrated a strong correlation in the UK Biobank ($r = 0.84$, Figure S2B).

Table S1. Biomarkers included in the biological age algorithm and their field IDs in UK Biobank.

Variables	Field ID	Included in KDM-BA	Included in PhenoAge
FEV1 (L)	3063	Yes	
SBP (mm Hg)	4080	Yes	
Total Cholesterol (mg/dL)	30690	Yes	
Glycated hemoglobin (%)	30750	Yes	
Blood urea nitrogen (mg/dL)	30670	Yes	
Albumin (g/dL)	30600	Yes	Yes
Creatinine (mg/dL)	30700	Yes	Yes
C-reactive protein (mg/dL)	30710	Yes	Yes
Alkaline phosphatase (U/L)	30610	Yes	Yes
Lymphocyte (%)	30180		Yes
Mean cell volume (fL)	30270		Yes
Serum glucose (mmol/L)	30740		Yes
Red cell distribution width (%)	30070		Yes
White blood cell count (1000 cells/μL)	30000		Yes

Abbreviations: KDM-BA, Klemera-Doubal Method Biological Age; PhenoAge, Phenotypic Age; FEV1, forced expiratory volume in 1-second; SBP, systolic blood pressure.

Table S2. Associations of biological age measures with mortality in NHANES IV.

Characteristic	Events/Participants	Person-years	Model 1	
			HR (95% CI)	*P*-value
**KDM-BA acceleration**				
Quartile				
Q1	35/2061	169281	1 (Reference)	
Q2	49/2061	167731	1.57 (1.02-2.42)	0.042
Q3	33/2060	167781	1.13 (0.7-1.82)	0.613
Q4	73/2061	165242	2.15 (1.43-3.21)	<0.001
P for trend				<0.001
Per SD increment			1.35 (1.19-1.53)	<0.001
**PhenoAge acceleration**				
Quartile				
Q1	497/9396	965844	1 (Reference)	
Q2	501/9396	887898	1.27 (1.12-1.44)	<0.001
Q3	543/9395	779021	1.65 (1.46-1.86)	<0.001
Q4	808/9396	620244	2.99 (2.66-3.35)	<0.001
P for trend				<0.001
Per SD increment			1.51 (1.46-1.56)	<0.001

Model 1: Adjusted for age and sex.

Abbreviations: HR, hazard ratio; CI, confidence interval; KDM-BA, Klemera-Doubal Method Biological Age; PhenoAge, Phenotypic Age; SD, standard deviation; Q, Quartile.

Table S3. Information of genetic variants associated with stroke in the UK Biobank.

SNP	Gene	Effect allele	Other allele	Beta	P value	Effect allele frequency
rs7917880	SH3PXD2A	C	A	0.0427	5.26E-06	0.4238
rs77559139		A	G	0.1371	5.27E-07	0.0369
rs2284665	HTRA1	G	T	0.0602	5.99E-08	0.7855
rs10883926	SH3PXD2A	A	G	0.0503	8.15E-08	0.6028
rs36053597		T	C	0.061	4.55E-07	0.1688
rs7124178	DENND5A	C	T	0.0524	8.98E-07	0.2477
rs12361415		T	G	0.0458	9.53E-06	0.7194
rs475937	WTAPP1	A	C	0.0757	2.92E-08	0.1318
rs60102266		C	T	0.0954	2.09E-06	0.0565
rs4556502	LOC105376567	A	T	0.1383	1.23E-06	0.0325
rs79280766		A	G	0.1434	2.20E-06	0.0362
rs7294375		G	T	0.053	3.33E-07	0.2711
rs11045896	LOC124902895	A	C	0.0487	7.44E-06	0.769
rs7488386	LOC643339	G	A	0.0412	9.15E-06	0.4369
rs9668073		T	G	0.0431	3.97E-06	0.5857
rs4140660	LOC105369612	T	C	0.0641	5.34E-06	0.1287
rs10774624	LINC02356	G	A	0.0654	4.04E-12	0.4715
rs4942561	rs4942561	T	G	0.064	2.05E-09	0.7581
rs74808730		G	A	0.0828	8.69E-06	0.9165
rs650724	COL4A1	G	A	0.0793	2.17E-06	0.9062
rs9521635	COL4A1	C	T	0.0423	9.36E-06	0.3665
rs35046086	LOC105370286	A	G	0.2006	7.19E-06	0.0144
rs4903725	NRXN3	T	C	0.0586	9.27E-06	0.8553
rs59844791		G	A	0.0633	8.15E-06	0.8386
rs4886564		C	T	0.0653	2.37E-06	0.1299
rs7200604	HS3ST4	T	C	0.0533	8.76E-06	0.664
rs9936039	CDH13	C	A	0.0561	1.98E-07	0.7494
rs879324	ZFHX3	A	G	0.0567	1.90E-06	0.1792
rs34416434	ZFHX3	C	T	0.1155	1.39E-06	0.9528
rs12445022	LOC124903748	A	G	0.052	1.03E-07	0.3346
rs7219031	RNF43	A	G	0.0596	2.59E-07	0.1933
rs111254932		A	C	0.1238	3.65E-06	0.0336
rs149520112	SMARCE1	T	G	0.1794	8.79E-06	0.024
rs117426542		A	G	0.1487	1.56E-06	0.0309
rs12326831	ZBTB7C	G	A	0.0857	7.93E-06	0.0622
rs34212978	PHLPP1	C	T	0.0687	5.79E-06	0.1041
rs1495900	FHOD3	G	A	0.0479	5.97E-06	0.258
rs8103309		T	C	0.0522	3.70E-07	0.6554
rs7250421	SLC44A2	C	T	0.0501	9.15E-06	0.7859
rs7538546	LOC105373220	T	C	0.0862	6.29E-06	0.0783
rs74976226	SMYD3	C	T	0.1435	9.66E-06	0.9466
rs1537407	PRDM16	C	T	0.0662	4.48E-07	0.2016

rs72699046	WNT2B	C	G	0.0897	1.53E-07	0.0824
rs2455134	PRDM16	G	A	0.0564	8.58E-06	0.7461
rs12562305	FGGY	A	G	0.0882	2.60E-06	0.0722
rs159963	RERE	A	C	0.0424	8.43E-06	0.5626
rs11587860		G	C	0.0689	2.54E-12	0.6455
rs7549874	PPFIA4	T	C	0.0495	2.07E-06	0.2868
rs17035646	CASZ1	A	G	0.0522	6.12E-08	0.3514
rs9613666	CHEK2	T	C	0.0426	6.80E-06	0.3693
rs72889922	ITGA6	A	G	0.2504	2.91E-06	0.015
rs6544653	THADA	C	T	0.0451	8.84E-06	0.6596
rs57269940	NRP2	T	C	0.0499	3.85E-06	0.2773
rs9852938		A	G	0.0586	9.41E-06	0.1396
rs12630390	LOC124909441	A	G	0.1184	3.72E-06	0.0407
rs10937513		A	C	0.0989	5.02E-07	0.061
rs28441665		C	T	0.0852	2.35E-06	0.072
rs7435890	ANK2	A	G	0.057	8.71E-08	0.302
rs6838973		C	T	0.0421	6.48E-06	0.5647
rs151080514	SMARCAD1-DT	T	C	0.2419	5.58E-06	0.0173
rs2634074		T	A	0.084	6.56E-14	0.2115
rs2066864	FGG	A	G	0.0562	1.29E-07	0.2455
rs11957829	LOC100505841	A	G	0.0616	1.51E-06	0.8315
rs6872625		T	G	0.051	4.21E-06	0.2381
rs79332111		C	T	0.0661	4.85E-06	0.1195
rs148159238		T	C	0.1486	8.86E-06	0.9762
rs11952498		T	C	0.0431	5.69E-06	0.4475
rs2060213	LOC124901196	C	T	0.0523	1.28E-06	0.2444
rs2585193	MEGF10	A	G	0.0504	2.45E-07	0.6842
rs147076266		G	A	0.2051	3.56E-06	0.0155
rs35276016	LINC02828	T	C	0.0984	9.31E-08	0.0851
rs4151702	CDKN1A DINOL	G	C	0.0551	2.63E-06	0.8
rs9502286	FARS2	A	T	0.1064	6.92E-06	0.9555
rs11242678	LINC01394	T	C	0.0643	8.71E-10	0.2551
rs1563788	ZNF318	T	C	0.0449	6.14E-06	0.2981
rs9386182	STXBP5	T	A	0.0467	4.22E-06	0.5032
rs2107595		A	G	0.0803	3.59E-11	0.1671
rs1549758	NOS3	T	C	0.0532	3.11E-07	0.3125
rs42039	CDK6	C	T	0.0574	1.65E-07	0.7545
rs75630045		A	G	0.2002	3.29E-06	0.0144
rs13312215		G	A	0.1103	9.26E-06	0.9284
rs147410905		G	A	0.1325	1.89E-06	0.0386
rs2978551	CLVS1	T	C	0.0446	3.39E-06	0.339
rs76893867	DLGAP2	C	G	0.0506	8.07E-06	0.3492
rs1537375	CDKN2B-AS1	C	T	0.0519	1.24E-08	0.5021

rs635634		T	C	0.0685	6.03E-08	0.1907
rs147861947	PALM2AKAP2	A	G	0.1342	4.51E-06	0.9631

Table S4. Components of Dietary Recommendations for Adherence to AHA Guidelines in the UK Biobank

Diet component	Field IDs	Amount per serving	Intake goal
Fruit	1309 (pieces fresh fruit/day)<br>1319 (pieces dried fruit/day)	1309 – 1 piece<br>1319 – 5 pieces	3 servings/day
Vegetable	1289 (tablespoons cooked vegetables/day)<br>1299 (salad/raw vegetables/day)	3 heaped tablespoons	3 servings/day
Whole grains	1438, 1448 (whole meal/wholegrain bread slices/week)<br>1458, 1468 (bran/oat/muesli cereal bowls/week)	1438/1448 – 1 slice/day<br>1458/1468 – 1 bowl/day	3 servings/day
(Shell)fish	1329 (oily fish/week)<br>1339 (non-oily fish/week)	Once/week	⩾2 servings/week
Dairy	1408 (cheese/week)<br>1418 (milk type)	1408 – 1 piece/day<br>1418 – 1 glass/day if consumption of any type of milk	2 servings/day
Vegetable oils	1428 (Flora Pro-Active/Benecol spread)<br>2654 (Flora Pro-Active/Benecol, soft margarine -, olive oil based -, polyunsaturated/sunflower oil based -, other low/reduced fat spread)<br>1438 (bread slices/week)	1 serving/day if in combination with eating at least 2 slices of bread (ID 1438)	2 servings/day
Refined grains	1438, 1448 (white, brown, other bread slices/week)<br>1458, 1468 (biscuit, other cereals/week)	1438/1448 – 1 slice/day<br>1458/1468 – 1 bowl/day	⩽2 servings/day

Processed meats	1349 (processed meat/week or daily)<br>3680 (age when last ate meat)	1349 – 1 piece/day<br>3680 – 0 pieces/day if indicated having never eaten meat	≤1 serving/week
Unprocessed meats	1359 (poultry/week or day)<br>1369 (beef/week or day)<br>1379 (lamb or mutton/week or day)<br>1389 (pork/week or day)<br>3680 (age when last ate meat)	1359-1389 – once/week<br>3680 – 0 pieces/day if indicated having never eaten meat	≤2 servings/week
Sugar-sweetened beverages	6144 (never consumes drinks containing sugar)	Only 0 servings were possible here.	Don’t drink

Field IDs and serving sizes used per diet component in UK Biobank with available data from the general baseline questionnaire. If participants achieved the intake goal, they were considered to have an adequate intake of the diet component.

Scoring criteria for dietary recommendations: 1: If intake goal met; 0: If intake goal not met. (Range: 0-10)

Table S5. Methods for evaluating each individual healthy behavior in the UK Biobank.

healthy behavior metric	Quantification of healthy behavior metric	
	Status	Scoring points
Diet	7-10 diet scores - 95th percentile (top/ideal diet)	100
	5-6 diet scores- 75th–94th percentile	80
	3-4 diet scores- 50th–74th percentile	50
	2 diet scores- 25th–49th percentile	25
	0-1 diet scores- 1st–24th percentile (bottom/ least ideal quartile)	0
Physical activity	⩾150 minutes	100
	120–149 minutes	90
	90–119 minutes	80
	60–89 minutes	60
	30–59 minutes	40
	1–29 minutes	20
	0 minutes	0
Nicotine exposure	Never smoker	100
	Former smoker, quit ≥ 5 years	75
	Former smoker, quit 1–<5 years	50
	Former smoker, quit <1 years	25

	Current smoker	0
	Subtract 20 points (unless score is 0) for living with active indoor smoker in home. Moreover, because the information on the specific time to quit smoking was only available in participants who indicated “smoked on most or all days in the past”. We regard the participants who indicated “smoked occasionally in the past” as equivalent to “Former smoker, quit 1–<5 years”; We regard the participants who indicated “just tried once or twice in the past” as equivalent to “Former smoker, quit ≥ 5 years”.	
Sleep health	7–<9 hours	100
	9–<10 hours	90
	6–<7 hours	70
	5–<6 or ≥10 hours	40
	4–<5 hours	20
	<4 hours	0
BMI	<25.0 kg/m2	100
	25.0–29.9 kg/m2	70
	30.0–34.9 kg/m2	30
	35.0–39.9 kg/m2	15
	≥40.0 kg/m2	0

Table S6. Detailed codes from ICD-10, ICD-9, and self-reported used to identify participants with stroke and its subtypes.

Diseases	ICD-9 diagnosis	ICD-10 diagnosis	Self-reported code
Stroke	430, 4309, 431, 4319, 434, 4340, 4341, 4349, 436, 4369	I60, I60.0, I60.1, I60.2, I60.3, I60.4, I60.5, I60.6, I60.7, I60.8, I60.9, I61, I61.0, I61.1, I61.2, I61.3, I61.4, I61.5, I61.6, I61.8, I61.9, I63, I63.0, I63.1, I63.2, I63.3, I63.4, I63.5, I63.6, I63.8, I63.9, I64	1081, 1086, 1491, 1583
Ischemic stroke	434, 4340, 4341, 4349, 436, 4369	I63, I63.0, I63.1, I63.2, I63.3, I63.4, I63.5, I63.6, I63.8, I63.9, I64	1583
Intracerebral hemorrhage	431, 4319	I61, I61.0, I61.1, I61.2, I61.3, I61.4, I61.5, I61.6, I61.8, I61.9	1491
Subarachnoid hemorrhage	430, 4309	I60.0, I60.1, I60.2, I60.3, I60.4, I60.5, I60.6, I60.7, I60.8, I60.9	1086

Abbreviations: ICD, International Classification of Disease.

Table S7. Association between polygenic risk score and risk of stroke and stroke subtypes.

Characteristic	Events/Participants	Person-years	Model 1		Model 2	
			HR (95% CI)	*P*-value	HR (95% CI)	*P*-value
**Stroke**						
**Polygenic risk score**						
Quintile						
Low (bottom quintile)	947/50797	674650	1 (Reference)		1 (Reference)	
Intermediate (quintiles 2-4)	3311/152359	2019530	1.18 (1.09-1.27)	<0.001	1.17 (1.09-1.26)	<0.001
High (top quintile)	1202/50776	673266	1.29 (1.18-1.4)	<0.001	1.28 (1.18-1.40)	<0.001
P for trend				<0.001		<0.001
Per SD increment			1.17 (1.12-1.23)	<0.001	1.17 (1.12-1.23)	<0.001
**Ischemic stroke**						
**Polygenic risk score**						
Quintile						
Low (bottom quintile)	746/50797	675369	1 (Reference)		1 (Reference)	
Intermediate (quintiles 2-4)	2637/152359	2021981	1.19 (1.1-1.29)	<0.001	1.19 (1.09-1.29)	<0.001
High (top quintile)	954/50776	674155	1.3 (1.18-1.43)	<0.001	1.29 (1.17-1.42)	<0.001
P for trend				<0.001		<0.001
Per SD increment			1.18 (1.12-1.25)	<0.001	1.18 (1.12-1.24)	<0.001
**Intracerebral hemorrhage**						
**Polygenic risk score**						
Quintile						
Low (bottom quintile)	159/50797	677998	1 (Reference)		1 (Reference)	
Intermediate (quintiles 2-4)	564/152359	2031084	1.19 (1-1.42)	0.052	1.19 (0.99-1.41)	0.057
High (top quintile)	228/50776	677405	1.45 (1.18-1.77)	<0.001	1.44 (1.18-1.77)	<0.001
P for trend				<0.001		<0.001
Per SD increment			1.22 (1.08-1.36)	0.001	1.21 (1.08-1.36)	0.001
**Subarachnoid hemorrhage**						

**Polygenic risk score**						
Quintile						
Low (bottom quintile)	112/50797	678037	1 (Reference)		1 (Reference)	
Intermediate (quintiles 2-4)	326/152359	2031290	0.97 (0.78-1.21)	0.801	0.97 (0.78-1.2)	0.771
High (top quintile)	115/50776	677572	1.03 (0.79-1.33)	0.834	1.03 (0.79-1.33)	0.849
P for trend				0.832		0.847
Per SD increment			1.05 (0.90-1.22)	0.509	1.05 (0.90-1.22)	0.520

Model 1: Adjusted for age and sex.

Model 2: Further adjusted for assessment center, household income, years of education, employment status, Index of Multiple Deprivation, alcohol consumption, behavior score, genotype batch, and the first ten genetic principal components.

The genetic risk was classified into low (bottom quintile), intermediate (quintiles 2-4), and high (top quintile) categories according to the distribution of PRSs.

Abbreviations: HR, hazard ratio; CI, confidence interval; SD, standard deviation.

Table S8. Additive interactions between biological age accelerations and genetic score and the risk of stroke and stroke subtypes.

Variable	Medium genetic risk		High genetic risk	
	RERI (95% CI)	AP (95% CI)	RERI (95% CI)	AP (95% CI)
**Stroke**				
**KDM-BA acceleration**				
Q1				
Q2	0.1(-0.13~0.33)	0.08(-0.1~0.26)	-0.18(-0.49~0.14)	-0.15(-0.41~0.11)
Q3	0.12(-0.12~0.35)	0.08(-0.09~0.25)	0.23(-0.08~0.54)	0.14(-0.04~0.32)
Q4	**0.27(0.02~0.52)**	**0.14(0.01~0.27)**	**0.35(0.02~0.68)**	**0.16(0.01~0.31)**
**PhenoAge acceleration**				
Q1				
Q2	-0.13(-0.4~0.13)	-0.11(-0.33~0.1)	-0.05(-0.38~0.28)	-0.03(-0.27~0.2)
Q3	0.02(-0.23~0.27)	0.01(-0.16~0.19)	0.01(-0.32~0.34)	0.01(-0.2~0.21)
Q4	-0.09(-0.37~0.2)	-0.05(-0.19~0.1)	-0.09(-0.46~0.28)	-0.04(-0.22~0.13)
**Ischemic stroke**				
**KDM-BA acceleration**				
Q1				
Q2	0.08(-0.19~0.36)	0.06(-0.14~0.25)	-0.14(-0.5~0.23)	-0.11(-0.39~0.18)
Q3	0.05(-0.24~0.34)	0.03(-0.16~0.22)	**0.37(0.01~0.73)**	**0.19(0.01~0.38)**
Q4	**0.3(0.01~0.59)**	**0.14(0~0.29)**	**0.54(0.15~0.93)**	**0.22(0.07~0.38)**
**PhenoAge acceleration**				
Q1				
Q2	-0.11(-0.42~0.19)	-0.09(-0.33~0.15)	0(-0.38~0.39)	0(-0.26~0.27)
Q3	0.06(-0.23~0.35)	0.04(-0.15~0.23)	0.12(-0.26~0.5)	0.07(-0.15~0.29)
Q4	-0.11(-0.44~0.23)	-0.05(-0.21~0.11)	0.02(-0.41~0.45)	0.01(-0.17~0.19)
**Intracerebral hemorrhage**				
**KDM-BA acceleration**				
Q1				

Q2	0.23(-0.22~0.68)	0.21(-0.23~0.64)	-0.11(-0.79~0.57)	-0.09(-0.66~0.48)
Q3	0.23(-0.24~0.71)	0.19(-0.22~0.6)	0(-0.69~0.7)	0(-0.48~0.49)
Q4	-0.13(-0.74~0.48)	-0.09(-0.48~0.31)	-0.31(-1.11~0.5)	-0.18(-0.64~0.29)
**PhenoAge acceleration**				
Q1				
Q2	0.19(-0.26~0.63)	0.19(-0.29~0.68)	0.05(-0.61~0.72)	0.04(-0.47~0.54)
Q3	0.25(-0.2~0.7)	0.22(-0.2~0.63)	0(-0.67~0.67)	0(-0.49~0.49)
Q4	-0.07(-0.62~0.47)	-0.05(-0.46~0.35)	-0.61(-1.42~0.2)	-0.46(-1.07~0.14)
**Subarachnoid hemorrhage**				
**KDM-BA acceleration**				
Q1				
Q2	0.26(-0.23~0.76)	0.32(-0.34~0.98)	-0.09(-0.81~0.63)	-0.11(-0.98~0.76)
Q3	0.37(-0.1~0.83)	0.43(-0.18~1.04)	-0.23(-0.93~0.48)	-0.4(-1.66~0.86)
Q4	**0.58(0.13~1.03)**	**0.51(0.05~0.98)**	0.46(-0.18~1.1)	0.36(-0.15~0.87)
**PhenoAge acceleration**				
Q1				
Q2	-0.6(-1.51~0.3)	-0.61(-1.41~0.19)	-0.1(-1.05~0.84)	-0.08(-0.78~0.63)
Q3	-0.76(-1.74~0.23)	-0.64(-1.36~0.08)	-0.51(-1.56~0.55)	-0.39(-1.21~0.43)
Q4	0.01(-0.74~0.76)	0.01(-0.48~0.5)	0.23(-0.66~1.11)	0.15(-0.42~0.72)

Adjusted for age, sex, assessment center, household income, years of education, employment status, Index of Multiple Deprivation, alcohol consumption, behavior score, genotype batch, and the first ten genetic principal components. To estimate RERI and AP, the lowest of biological age acceleration group and the lowest PRS group were set as reference.

Abbreviations: CI, confidence interval; KDM-BA, Klemera-Doubal Method Biological Age; PhenoAge, Phenotypic Age; Q, Quartile.

Table S9. Association between healthy behaviors score and risk of stroke and stroke subtypes.

Characteristic	Events/Participants	Person-years	Model 1		Model 2	
			HR (95% CI)	*P*-value	HR (95% CI)	*P*-value
**Stroke**						
**Health behaviors score**						
Tertiles						
Low	2159/84579	1111775	1 (Reference)		1 (Reference)	
Intermediate	1797/86297	1145403	0.76 (0.71-0.81)	<0.001	0.79 (0.74-0.84)	<0.001
High	1504/83056	1110267	0.67 (0.63-0.72)	<0.001	0.71 (0.67-0.76)	<0.001
P for trend				<0.001		<0.001
Per 10-point increment			0.87 (0.86-0.89)	<0.001	0.89 (0.88-0.91)	<0.001
**Ischemic stroke**						
**Health behaviors score**						
Tertiles						
Low	1760/84579	1113143	1 (Reference)		1 (Reference)	
Intermediate	1420/86297	1146760	0.73 (0.68-0.79)	<0.001	0.76 (0.71-0.82)	<0.001
High	1157/83056	1111602	0.64 (0.59-0.69)	<0.001	0.68 (0.63-0.73)	<0.001
P for trend				<0.001		<0.001
Per 10-point increment			0.86 (0.84-0.88)	<0.001	0.88 (0.86-0.9)	<0.001
**Intracerebral hemorrhage**						
**Health behaviors score**						
Tertiles						
Low	347/84579	1119346	1 (Reference)		1 (Reference)	
Intermediate	305/86297	1151622	0.8 (0.68-0.93)	0.004	0.81 (0.7-0.95)	0.009
High	299/83056	1115520	0.82 (0.7-0.95)	0.01	0.84 (0.72-0.98)	0.028
P for trend				0.009		0.026
Per 10-point increment			0.92 (0.87-0.96)	<0.001	0.93 (0.88-0.97)	0.001
**Subarachnoid hemorrhage**						

**Health behaviors score**						
Tertiles						
Low	199/84579	1119544	1 (Reference)		1 (Reference)	
Intermediate	193/86297	1151709	0.91 (0.75-1.11)	0.375	0.95 (0.78-1.16)	0.634
High	161/83056	1115646	0.76 (0.61-0.93)	0.009	0.81 (0.65-1.00)	0.049
P for trend				0.009		0.051
Per 10-point increment			0.92 (0.87-0.98)	0.011	0.95 (0.89-1.01)	0.076

Model 1: Adjusted for age, and sex.

Model 2: Further adjusted for assessment center, household income, years of education, employment status, Index of Multiple Deprivation, and alcohol consumption.

Abbreviations: HR, hazard ratio; CI, confidence interval.

Table S10. Association between biological age accelerations and risk of stroke and stroke subtypes after excluding participants diagnosed with stroke within the first two years of follow-up.

Characteristic	Events/Participants	Person-years	Model 1		Model 2	
			HR (95% CI)	*P*-value	HR (95% CI)	*P*-value
**Stroke**						
**KDM-BA acceleration**						
Quartile						
Q1	1037/63393	846193	1 (Reference)		1 (Reference)	
Q2	1137/63392	844908	1.22 (1.12-1.33)	<0.001	1.17 (1.08-1.28)	<0.001
Q3	1250/63392	843238	1.39 (1.28-1.51)	<0.001	1.3 (1.19-1.41)	<0.001
Q4	1674/63393	832731	1.95 (1.81-2.11)	<0.001	1.74 (1.61-1.89)	<0.001
P for trend				<0.001		<0.001
Per SD increment			1.33 (1.29-1.36)	<0.001	1.27 (1.24-1.31)	<0.001
**PhenoAge acceleration**						
Quartile						
Q1	1003/63393	853890	1 (Reference)		1 (Reference)	
Q2	1016/63392	849033	0.98 (0.9-1.07)	0.705	0.96 (0.88-1.04)	0.311
Q3	1291/63392	843048	1.22 (1.12-1.33)	<0.001	1.16 (1.06-1.26)	0.001
Q4	1788/63393	821097	1.7 (1.57-1.84)	<0.001	1.52 (1.4-1.65)	<0.001
P for trend				<0.001		<0.001
Per SD increment			1.26 (1.23-1.29)	<0.001	1.21 (1.18-1.25)	<0.001
**Ischemic stroke**						
**KDM-BA acceleration**						
Quartile						
Q1	794/63393	846849	1 (Reference)		1 (Reference)	
Q2	904/63392	845726	1.29 (1.17-1.42)	<0.001	1.24 (1.12-1.36)	<0.001
Q3	994/63392	844050	1.49 (1.35-1.64)	<0.001	1.37 (1.25-1.51)	<0.001
Q4	1360/63393	833740	2.15 (1.97-2.35)	<0.001	1.89 (1.72-2.07)	<0.001

P for trend				<0.001		<0.001
Per SD increment			1.37 (1.33-1.42)	<0.001	1.31 (1.27-1.35)	<0.001
**PhenoAge acceleration**						
Quartile						
Q1	734/63393	854788			1 (Reference)	
Q2	794/63392	849735	1.03 (0.94-1.14)	0.513	1 (0.91-1.11)	0.977
Q3	1038/63392	843748	1.31 (1.19-1.44)	<0.001	1.23 (1.12-1.36)	<0.001
Q4	1486/63393	822093	1.87 (1.71-2.05)	<0.001	1.65 (1.5-1.81)	<0.001
P for trend				<0.001		<0.001
Per SD increment			1.31 (1.27-1.34)	<0.001	1.25 (1.22-1.29)	<0.001
**Intracerebral hemorrhage**						
**KDM-BA acceleration**						
Quartile						
Q1	215/63393	849221	1 (Reference)		1 (Reference)	
Q2	193/63392	848510	0.96 (0.79-1.17)	0.698	0.95 (0.78-1.15)	0.572
Q3	214/63392	847351	1.08 (0.9-1.31)	0.406	1.05 (0.86-1.27)	0.629
Q4	268/63393	837967	1.4 (1.17-1.69)	<0.001	1.32 (1.09-1.6)	0.004
P for trend				<0.001		0.002
Per SD increment			1.17 (1.1-1.25)	<0.001	1.15 (1.07-1.23)	<0.001
**PhenoAge acceleration**						
Quartile						
Q1	219/63393	856868	1 (Reference)		1 (Reference)	
Q2	178/63392	852064	0.81 (0.66-0.98)	0.034	0.79 (0.65-0.97)	0.022
Q3	227/63392	847311	1.01 (0.84-1.22)	0.89	0.98 (0.81-1.18)	0.828
Q4	266/63393	826806	1.19 (0.99-1.43)	0.057	1.11 (0.92-1.34)	0.276
P for trend				0.009		0.077
Per SD increment			1.1 (1.03-1.18)	0.003	1.07 (1-1.14)	0.048
**Subarachnoid hemorrhage**						

**KDM-BA acceleration**						
Quartile						
Q1	101/63393	849393	1 (Reference)		1 (Reference)	
Q2	119/63392	848629	1.13 (0.86-1.47)	0.385	1.1 (0.85-1.44)	0.468
Q3	112/63392	847391	1.03 (0.79-1.35)	0.825	0.99 (0.75-1.31)	0.961
Q4	166/63393	838048	1.52 (1.18-1.95)	0.001	1.41 (1.09-1.83)	0.009
P for trend				0.002		0.016
Per SD increment			1.19 (1.09-1.3)	<0.001	1.16 (1.06-1.27)	0.002
**PhenoAge acceleration**						
Quartile						
Q1	122/63393	857027	1 (Reference)		1 (Reference)	
Q2	109/63392	852079	0.96 (0.74-1.25)	0.778	0.95 (0.73-1.23)	0.683
Q3	128/63392	847423	1.17 (0.91-1.51)	0.209	1.13 (0.88-1.46)	0.331
Q4	139/63393	826933	1.33 (1.04-1.71)	0.022	1.24 (0.96-1.6)	0.102
P for trend				0.008		0.049
Per SD increment			1.1 (1.01-1.2)	0.023	1.07 (0.98-1.17)	0.139

Model 1: Adjusted for age and sex.
Model 2: Further adjusted for assessment center, household income, years of education, employment status, Index of Multiple Deprivation, alcohol consumption, and behaviors score.
Abbreviations: HR, hazard ratio; CI, confidence interval; KDM-BA, Klemera-Doubal Method Biological Age; PhenoAge, Phenotypic Age; SD, standard deviation; Q, Quartile.

Table S11. Association between biological age accelerations and risk of stroke and stroke subtypes for nine biomarkers with only one missing value imputed using the median.

Characteristic	Events/Participants	Person-years	Model 1		Model 2	
			HR (95% CI)	*P*-value	HR (95% CI)	*P*-value
**Stroke**						
**KDM-BA acceleration**						
Quartile						
Q1	1383/77129	1028448	1 (Reference)		1 (Reference)	
Q2	1504/77129	1027056	1.19 (1.1-1.28)	<0.001	1.14 (1.06-1.22)	0.001
Q3	1667/77129	1024968	1.36 (1.26-1.46)	<0.001	1.26 (1.17-1.35)	<0.001
Q4	2252/77129	1010131	1.93 (1.8-2.07)	<0.001	1.71 (1.59-1.83)	<0.001
P for trend				<0.001		<0.001
Per SD increment			1.32 (1.29-1.35)	<0.001	1.26 (1.23-1.3)	<0.001
**PhenoAge acceleration**						
Quartile						
Q1	1324/77129	1038993	1 (Reference)		1 (Reference)	
Q2	1352/77129	1032670	0.99 (0.92-1.07)	0.787	0.96 (0.89-1.04)	0.287
Q3	1687/77129	1024881	1.21 (1.12-1.3)	<0.001	1.14 (1.06-1.22)	0.001
Q4	2443/77129	994058	1.76 (1.64-1.88)	<0.001	1.56 (1.45-1.67)	<0.001
P for trend				<0.001		<0.001
Per SD increment			1.28 (1.26-1.31)	<0.001	1.23 (1.21-1.26)	<0.001
**Ischemic stroke**						
**KDM-BA acceleration**						
Quartile						
Q1	1054/77129	1029503	1 (Reference)		1 (Reference)	
Q2	1200/77129	1028153	1.26 (1.16-1.37)	<0.001	1.2 (1.11-1.31)	<0.001
Q3	1312/77129	1026280	1.43 (1.32-1.56)	<0.001	1.32 (1.21-1.43)	<0.001
Q4	1829/77129	1011675	2.12 (1.97-2.29)	<0.001	1.85 (1.71-2.01)	<0.001

P for trend				<0.001		<0.001
Per SD increment			1.37 (1.33-1.4)	<0.001	1.3 (1.27-1.34)	<0.001
**PhenoAge acceleration**						
Quartile						
Q1	976/77129	1040264	1 (Reference)		1 (Reference)	
Q2	1040/77129	1033771	1.02 (0.93-1.11)	0.711	0.98 (0.9-1.07)	0.698
Q3	1360/77129	1025906	1.29 (1.18-1.4)	<0.001	1.21 (1.11-1.31)	<0.001
Q4	2019/77129	995670	1.91 (1.77-2.07)	<0.001	1.67 (1.54-1.81)	<0.001
P for trend				<0.001		<0.001
Per SD increment			1.32 (1.29-1.35)	<0.001	1.27 (1.24-1.3)	<0.001
**Intracerebral hemorrhage**						
**KDM-BA acceleration**						
Quartile						
Q1	286/77129	1032959	1 (Reference)		1 (Reference)	
Q2	247/77129	1032305	0.91 (0.77-1.08)	0.271	0.89 (0.75-1.06)	0.18
Q3	283/77129	1030957	1.06 (0.9-1.25)	0.514	1.01 (0.86-1.2)	0.864
Q4	361/77129	1018245	1.4 (1.2-1.64)	<0.001	1.31 (1.11-1.54)	0.001
P for trend				<0.001		<0.001
Per SD increment			1.18 (1.12-1.25)	<0.001	1.15 (1.09-1.22)	<0.001
**PhenoAge acceleration**						
Quartile						
Q1	277/77129	1043427	1 (Reference)		1 (Reference)	
Q2	247/77129	1037161	0.89 (0.75-1.06)	0.178	0.87 (0.73-1.03)	0.116
Q3	286/77129	1031110	1.02 (0.86-1.2)	0.861	0.98 (0.83-1.16)	0.803
Q4	367/77129	1002768	1.32 (1.12-1.55)	0.001	1.22 (1.03-1.44)	0.019
P for trend				<0.001		0.005
Per SD increment			1.16 (1.1-1.22)	<0.001	1.13 (1.07-1.19)	<0.001
**Subarachnoid hemorrhage**						

**KDM-BA acceleration**						
Quartile						
Q1	145/77129	1033153	1 (Reference)		1 (Reference)	
Q2	159/77129	1032457	1.06 (0.85-1.33)	0.605	1.03 (0.82-1.29)	0.784
Q3	172/77129	1030966	1.13 (0.91-1.41)	0.276	1.07 (0.86-1.35)	0.533
Q4	216/77129	1018374	1.43 (1.16-1.77)	0.001	1.3 (1.05-1.62)	0.018
P for trend				0.001		0.014
Per SD increment			1.17 (1.09-1.26)	<0.001	1.13 (1.05-1.22)	0.002
**PhenoAge acceleration**						
Quartile						
Q1	168/77129	1043517	1 (Reference)		1 (Reference)	
Q2	159/77129	1037237	1.01 (0.81-1.25)	0.949	0.98 (0.79-1.22)	0.875
Q3	164/77129	1031140	1.07 (0.86-1.33)	0.537	1.02 (0.82-1.27)	0.859
Q4	201/77129	1003056	1.37 (1.11-1.69)	0.003	1.24 (1-1.53)	0.053
P for trend				0.003		0.049
Per SD increment			1.1 (1.02-1.18)	0.009	1.06 (0.98-1.14)	0.156

Model 1: Adjusted for age and sex.

Model 2: Further adjusted for assessment center, household income, years of education, employment status, Index of Multiple Deprivation, alcohol consumption, and behaviors score.

Abbreviations: HR, hazard ratio; CI, confidence interval; KDM-BA, Klemera-Doubal Method Biological Age; PhenoAge, Phenotypic Age; SD, standard deviation; Q, Quartile.

Table S12. Association between biological age accelerations and risk of stroke and stroke subtypes using a data set without missing covariates.

Characteristic	Events/Participants	Person-years	Model 1		Model 2	
			HR (95% CI)	*P*-value	HR (95% CI)	*P*-value
**Stroke**						
**KDM-BA acceleration**						
Quartile						
Q1	924/52260	696579	1 (Reference)		1 (Reference)	
Q2	994/52259	695881	1.19 (1.09-1.3)	<0.001	1.15 (1.04-1.27)	0.006
Q3	1095/52259	694150	1.36 (1.25-1.49)	<0.001	1.26 (1.15-1.39)	<0.001
Q4	1486/52260	685138	1.93 (1.78-2.1)	<0.001	1.74 (1.58-1.91)	<0.001
P for trend				<0.001		<0.001
Per SD increment			1.33 (1.29-1.36)	<0.001	1.28 (1.24-1.32)	<0.001
**PhenoAge acceleration**						
Quartile						
Q1	884/52260	703234	1 (Reference)		1 (Reference)	
Q2	885/52259	699254	0.97 (0.89-1.07)	0.551	0.96 (0.86-1.06)	0.435
Q3	1132/52259	694207	1.21 (1.11-1.33)	<0.001	1.16 (1.05-1.28)	0.004
Q4	1598/52260	675052	1.72 (1.58-1.87)	<0.001	1.55 (1.41-1.71)	<0.001
P for trend				<0.001		<0.001
Per SD increment			1.28 (1.24-1.31)	<0.001	1.22 (1.19-1.26)	<0.001
**Ischemic stroke**						
**KDM-BA acceleration**						
Quartile						
Q1	705/52260	697265	1 (Reference)		1 (Reference)	
Q2	793/52259	696657	1.27 (1.15-1.41)	<0.001	1.21 (1.08-1.36)	0.001
Q3	858/52259	695033	1.44 (1.3-1.59)	<0.001	1.32 (1.18-1.48)	<0.001
Q4	1202/52260	686192	2.12 (1.93-2.33)	<0.001	1.88 (1.69-2.09)	<0.001

P for trend				<0.001		<0.001
Per SD increment			1.37 (1.33-1.41)	<0.001	1.31 (1.26-1.36)	<0.001
**PhenoAge acceleration**						
Quartile						
Q1	658/52260	704060	1 (Reference)		1 (Reference)	
Q2	690/52259	699978	1 (0.9-1.12)	0.943	1 (0.88-1.12)	0.952
Q3	907/52259	694936	1.28 (1.15-1.41)	<0.001	1.2 (1.08-1.35)	0.001
Q4	1303/52260	676173	1.83 (1.66-2.01)	<0.001	1.62 (1.45-1.81)	<0.001
P for trend				<0.001		<0.001
Per SD increment			1.31 (1.27-1.35)	<0.001	1.25 (1.21-1.29)	<0.001
**Intracerebral hemorrhage**						
**KDM-BA acceleration**						
Quartile						
Q1	197/52260	699550	1 (Reference)		1 (Reference)	
Q2	169/52259	699403	0.92 (0.75-1.13)	0.445	0.83 (0.66-1.04)	0.108
Q3	192/52259	698192	1.07 (0.87-1.31)	0.51	0.98 (0.79-1.23)	0.886
Q4	233/52260	690457	1.34 (1.1-1.62)	0.003	1.26 (1.01-1.57)	0.037
P for trend				0.001		0.015
Per SD increment			1.17 (1.09-1.25)	<0.001	1.16 (1.07-1.26)	<0.001
**PhenoAge acceleration**						
Quartile						
Q1	185/52260	706248	1 (Reference)		1 (Reference)	
Q2	160/52259	702206	0.85 (0.69-1.06)	0.148	0.85 (0.67-1.08)	0.172
Q3	189/52259	698431	0.99 (0.81-1.22)	0.923	0.98 (0.78-1.23)	0.859
Q4	257/52260	680717	1.35 (1.12-1.64)	0.002	1.29 (1.03-1.61)	0.024
P for trend				<0.001		0.006
Per SD increment			1.16 (1.08-1.24)	<0.001	1.11 (1.03-1.2)	0.007
**Subarachnoid hemorrhage**						

**KDM-BA acceleration**						
Quartile						
Q1	95/52260	699675	1 (Reference)		1 (Reference)	
Q2	107/52259	699459	1.09 (0.83-1.44)	0.525	1.15 (0.85-1.56)	0.349
Q3	109/52259	698225	1.09 (0.83-1.44)	0.531	1.04 (0.76-1.42)	0.805
Q4	153/52260	690465	1.53 (1.18-1.98)	0.001	1.45 (1.08-1.95)	0.014
P for trend				0.001		0.027
Per SD increment			1.19 (1.08-1.3)	<0.001	1.16 (1.04-1.29)	0.006
**PhenoAge acceleration**						
Quartile						
Q1	106/52260	706325	1 (Reference)		1 (Reference)	
Q2	100/52259	702184	1.01 (0.76-1.32)	0.965	0.98 (0.72-1.33)	0.888
Q3	125/52259	698454	1.3 (1-1.68)	0.052	1.31 (0.98-1.75)	0.071
Q4	133/52260	680863	1.43 (1.11-1.86)	0.006	1.43 (1.06-1.92)	0.018
P for trend				0.001		0.004
Per SD increment			1.13 (1.03-1.23)	0.008	1.12 (1.01-1.23)	0.028

Model 1: Adjusted for age and sex.

Model 2: Further adjusted for assessment center, household income, years of education, employment status, Index of Multiple Deprivation, alcohol consumption, and behaviors score.

Abbreviations: HR, hazard ratio; CI, confidence interval; KDM-BA, Klemera-Doubal Method Biological Age; PhenoAge, Phenotypic Age; SD, standard deviation; Q, Quartile.

Table S13. Association between biological age accelerations and risk of stroke and stroke subtypes using competitive risk model.

Characteristic	Events/Participants	Person-years	Model 1		Model 2	
			HR (95% CI)	*P*-value	HR (95% CI)	*P*-value
**Stroke**						
**KDM-BA acceleration**						
Quartile						
Q1	1103/63483	846597	1 (Reference)		1 (Reference)	
Q2	1204/63483	845298	1.2 (1.11-1.31)	<0.001	1.16 (1.07-1.26)	<0.001
Q3	1339/63483	843369	1.38 (1.27-1.49)	<0.001	1.29 (1.19-1.4)	<0.001
Q4	1814/63483	832182	1.91 (1.77-2.06)	<0.001	1.71 (1.58-1.85)	<0.001
P for trend				<0.001		<0.001
Per SD increment			1.31 (1.27-1.34)	<0.001	1.26 (1.22-1.29)	<0.001
**PhenoAge acceleration**						
Quartile						
Q1	1063/63483	854349	1 (Reference)		1 (Reference)	
Q2	1082/63483	849453	0.98 (0.9-1.07)	0.68	0.96 (0.88-1.04)	0.3
Q3	1374/63483	843251	1.21 (1.12-1.32)	<0.001	1.15 (1.06-1.25)	0.001
Q4	1941/63483	820393	1.65 (1.53-1.78)	<0.001	1.48 (1.37-1.6)	<0.001
P for trend				<0.001		<0.001
Per SD increment			1.23 (1.2-1.26)	<0.001	1.19 (1.16-1.21)	<0.001
**Ischemic stroke**						
**KDM-BA acceleration**						
Quartile						
Q1	845/63483	847401	1 (Reference)		1 (Reference)	
Q2	957/63483	846260	1.27 (1.16-1.4)	<0.001	1.22 (1.11-1.34)	<0.001
Q3	1065/63483	844373	1.47 (1.34-1.61)	<0.001	1.36 (1.24-1.5)	<0.001
Q4	1470/63483	833472	2.09 (1.92-2.28)	<0.001	1.84 (1.68-2.01)	<0.001
P for trend				<0.001		<0.001

Per SD increment			1.35 (1.31-1.39)	<0.001	1.29 (1.25-1.33)	<0.001
**PhenoAge acceleration**						
Quartile						
Q1	782/63483	855379	1 (Reference)		1 (Reference)	
Q2	844/63483	850327	1.03 (0.93-1.13)	0.61	1 (0.9-1.1)	0.92
Q3	1107/63483	844098	1.3 (1.18-1.42)	<0.001	1.22 (1.11-1.34)	<0.001
Q4	1604/63483	821701	1.79 (1.64-1.96)	<0.001	1.59 (1.45-1.74)	<0.001
P for trend				<0.001		<0.001
Per SD increment			1.27 (1.24-1.3)	<0.001	1.22 (1.19-1.25)	<0.001
**Intracerebral hemorrhage**						
**KDM-BA acceleration**						
Quartile						
Q1	230/63483	850148	1 (Reference)		1 (Reference)	
Q2	205/63483	849469	0.95 (0.78-1.14)	0.57	0.93 (0.77-1.12)	0.44
Q3	226/63483	848244	1.06 (0.88-1.27)	0.57	1.02 (0.84-1.23)	0.88
Q4	290/63483	838627	1.36 (1.14-1.63)	0.001	1.27 (1.06-1.53)	0.011
P for trend				<0.001		0.007
Per SD increment			1.16 (1.09-1.24)	<0.001	1.13 (1.06-1.22)	<0.001
**PhenoAge acceleration**						
Quartile						
Q1	227/63483	857895	1 (Reference)		1 (Reference)	
Q2	194/63483	853038	0.84 (0.69-1.02)	0.083	0.83 (0.68-1)	0.054
Q3	238/63483	848278	1.01 (0.84-1.22)	0.89	0.98 (0.81-1.18)	0.81
Q4	292/63483	827277	1.2 (1-1.43)	0.048	1.11 (0.92-1.33)	0.28
P for trend				0.012		0.1
Per SD increment			1.09 (1.02-1.16)	0.01	1.05 (0.99-1.13)	0.12
**Subarachnoid hemorrhage**						
**KDM-BA acceleration**						

Quartile						
Q1	113/63483	850358	1 (Reference)		1 (Reference)	
Q2	129/63483	849576	1.09 (0.85-1.4)	0.5	1.07 (0.83-1.38)	0.58
Q3	125/63483	848276	1.03 (0.8-1.33)	0.83	0.99 (0.77-1.29)	0.97
Q4	186/63483	838688	1.5 (1.18-1.9)	0.001	1.4 (1.1-1.79)	0.007
P for trend				0.002		0.011
Per SD increment			1.18 (1.08-1.29)	<0.001	1.15 (1.06-1.26)	0.002
**PhenoAge acceleration**						
Quartile						
Q1	133/63483	858025	1 (Reference)		1 (Reference)	
Q2	120/63483	853084	0.96 (0.75-1.24)	0.77	0.95 (0.74-1.22)	0.68
Q3	141/63483	848361	1.17 (0.92-1.49)	0.2	1.13 (0.89-1.44)	0.32
Q4	159/63483	827429	1.34 (1.06-1.7)	0.016	1.25 (0.97-1.6)	0.08
P for trend				0.006		0.038
Per SD increment			1.1 (1.02-1.18)	0.016	1.07 (0.98-1.15)	0.12

Model 1: Adjusted for age and sex.

Model 2: Further adjusted for assessment center, household income, years of education, employment status, Index of Multiple Deprivation, alcohol consumption, and behaviors score.

Abbreviations: HR, hazard ratio; CI, confidence interval; KDM-BA, Klemera-Doubal Method Biological Age; PhenoAge, Phenotypic Age; SD, standard deviation; Q, Quartile.

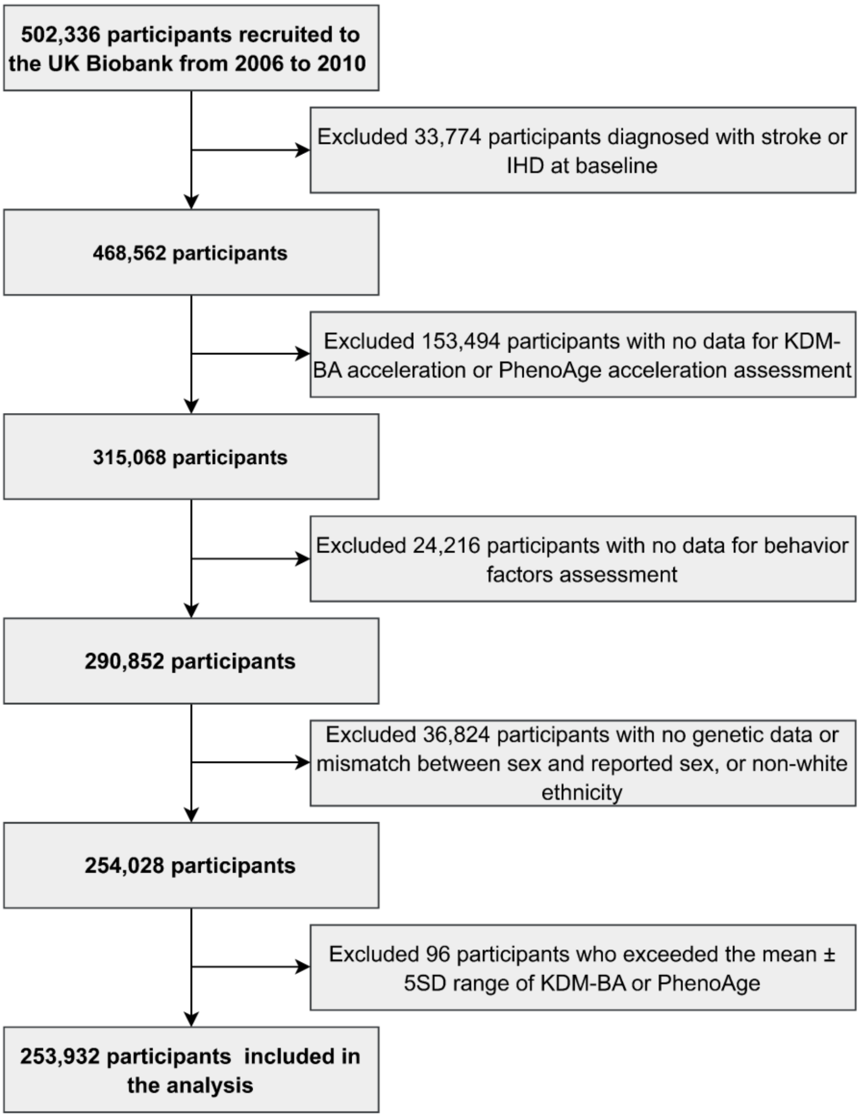

Figure S1. Flow diagram for inclusion of participants in this study.

Abbreviations: KDM-BA, Klemera-Doubal Method Biological Age; PhenoAge, Phenotypic Age; SD, standard deviation.

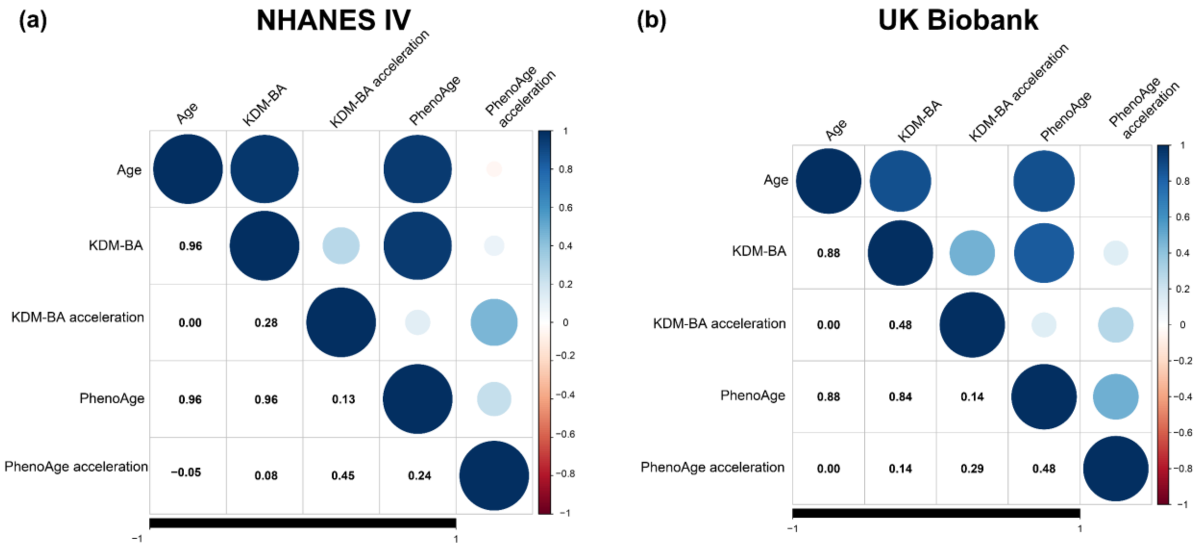

Figure S2. Correlations between chronological age, biological ages, and age accelerations in (a) NHANES IV and (b) UK Biobank.

Abbreviations: NHANES, National Health and Nutrition Examination Surveys; KDM-BA, Klemera-Doubal Method Biological Age; PhenoAge, Phenotypic Age.

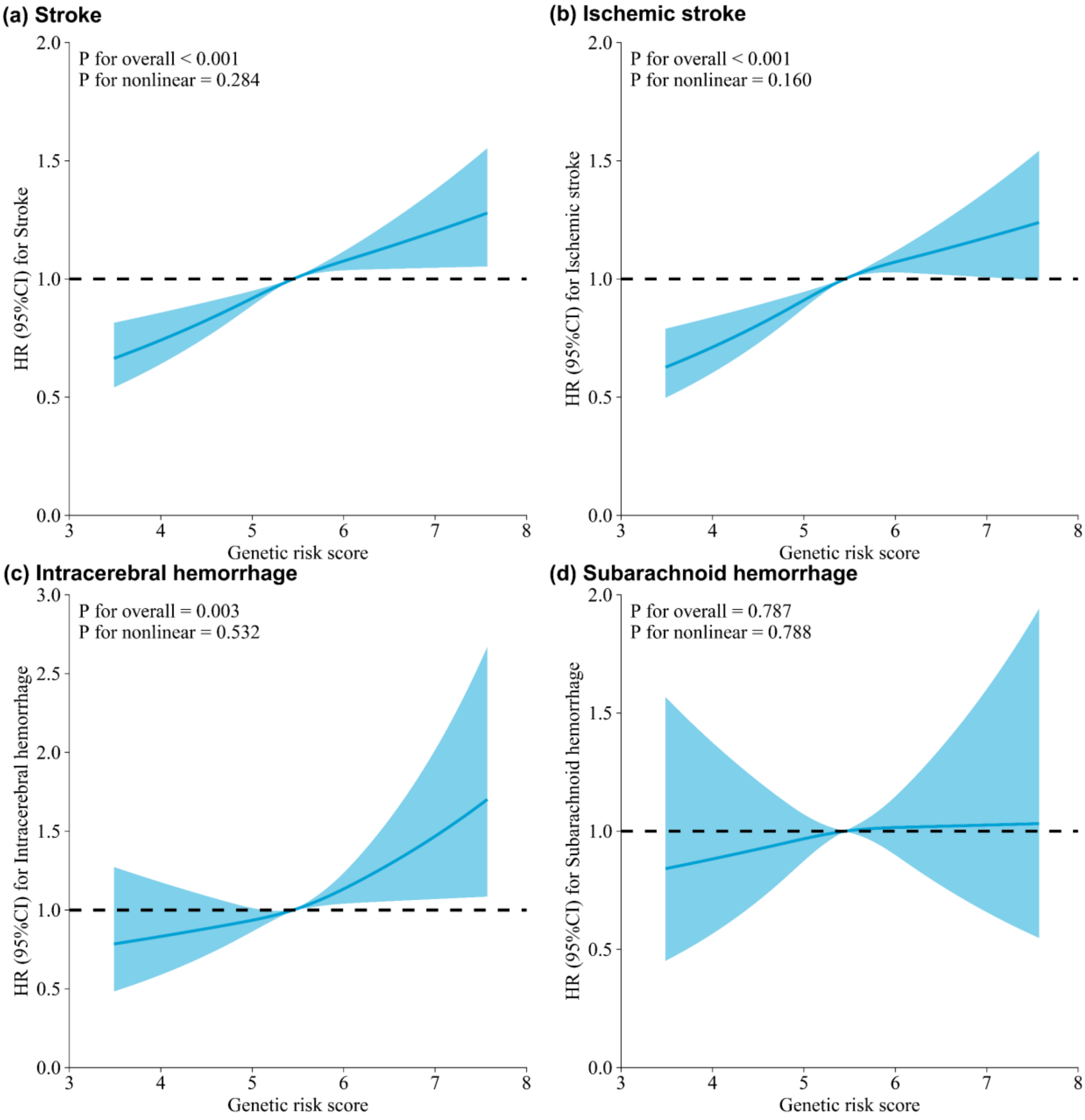

Figure S3. Association between genetic risk score and risk of stroke and stroke subtypes using restricted cubic splines models with three knots.

Models adjusted for age, sex, assessment center, household income, years of education, employment status, Index of Multiple Deprivation, alcohol consumption, behaviors score, genotype batch, and the first ten genetic principal components. Abbreviations: HR, hazard ratio; CI, confidence interval.

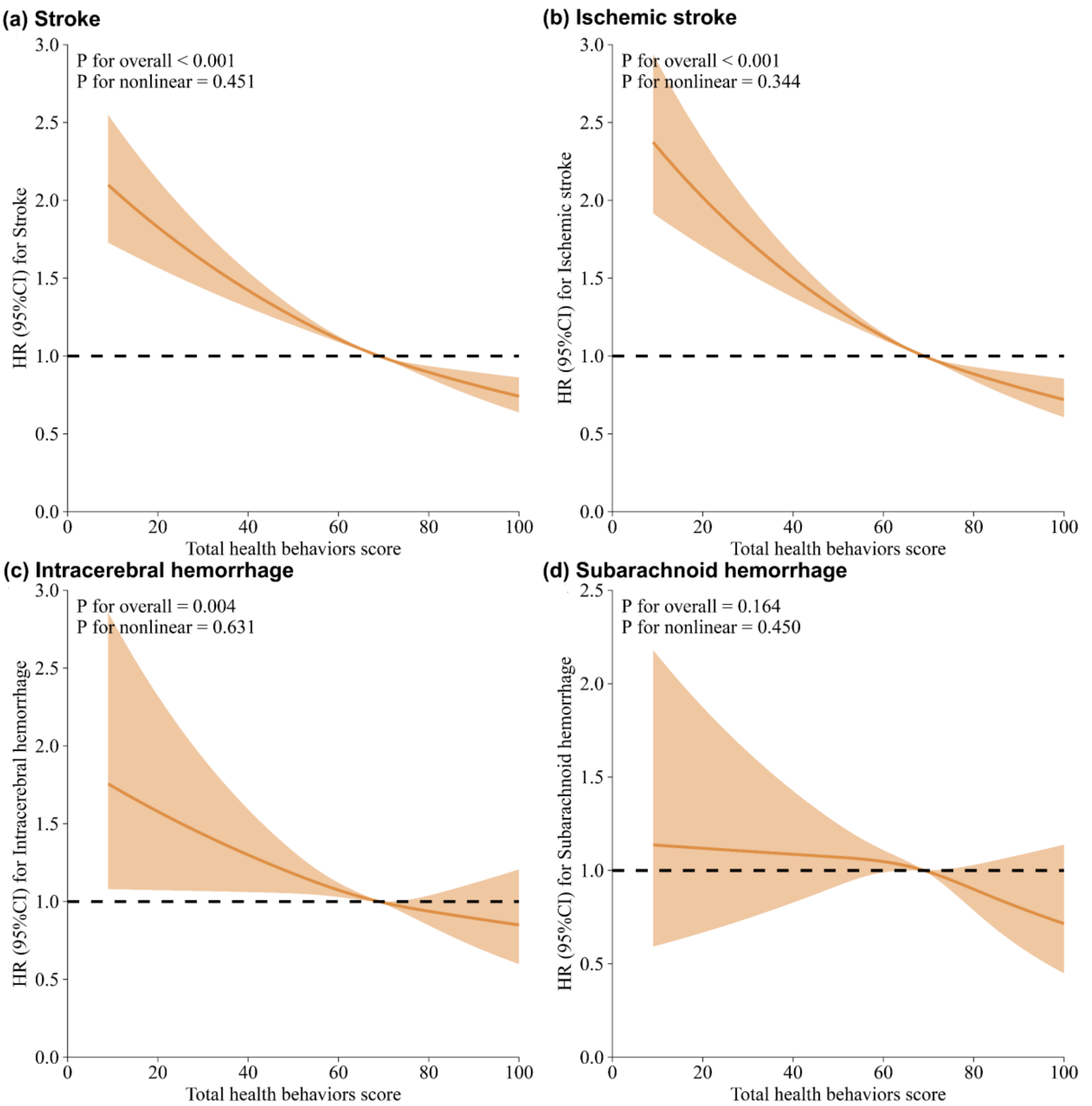

Figure S4. Association between total health behaviors score and risk of stroke and stroke subtypes using restricted cubic splines models with three knots.

Models adjusted for age, sex, assessment center, household income, years of education, employment status, Index of Multiple Deprivation, and alcohol consumption. Abbreviations: HR, hazard ratio; CI, confidence interval.

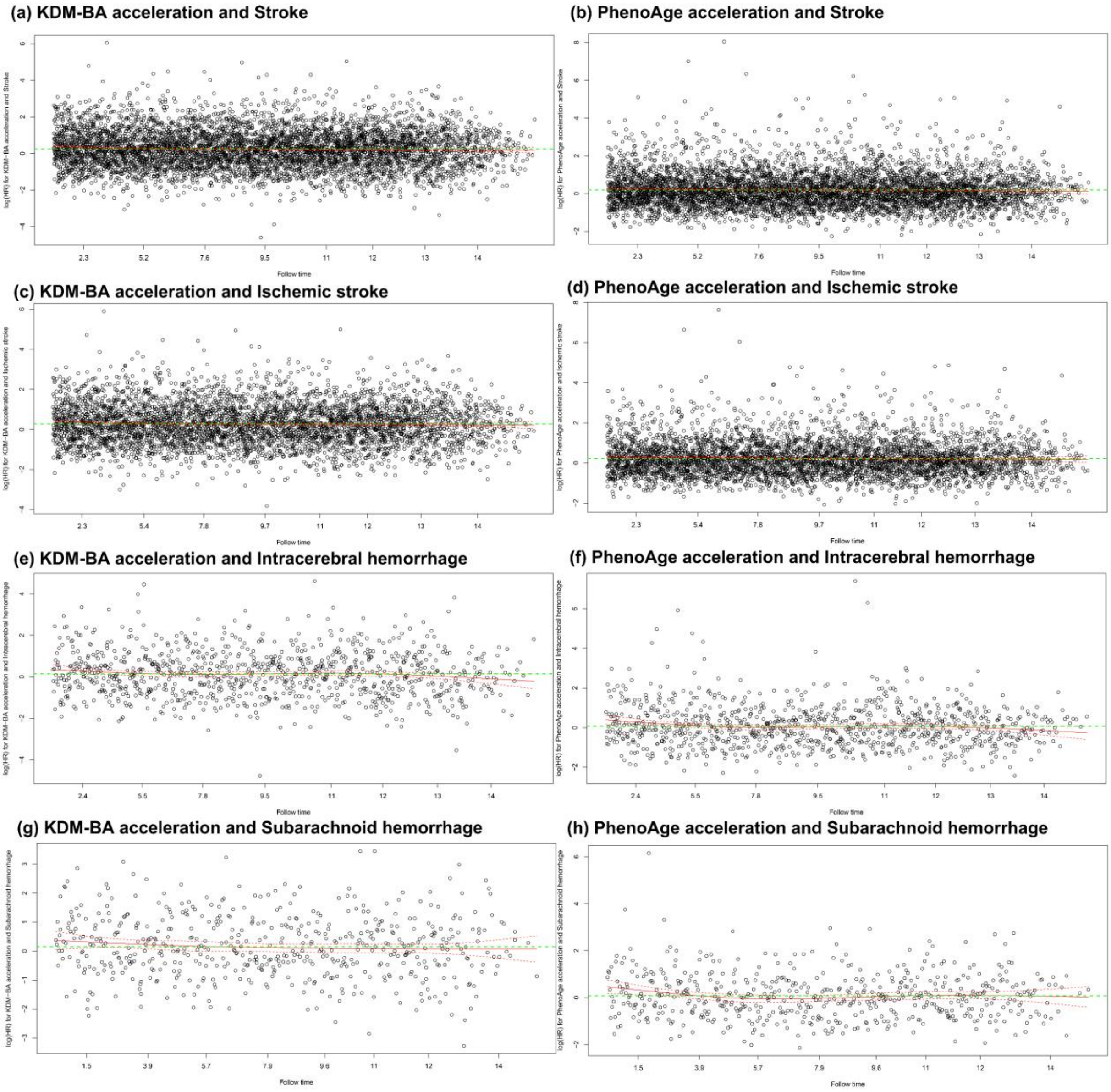

Figure S5. Scaling Schoenfeld residuals and time-based logarithmic HR plots of biological age acceleration for stroke and subtypes.

(a) KDM-BA acceleration and Stroke; (b) PhenoAge acceleration and Stroke; (c) KDM-BA acceleration and Ischemic stroke; (d) PhenoAge acceleration and Ischemic stroke; (e) KDM-BA acceleration and Intracerebral hemorrhage; (f) PhenoAge acceleration and Intracerebral hemorrhage; (g) KDM-BA acceleration and Subarachnoid hemorrhage; (h) PhenoAge acceleration and Subarachnoid hemorrhage. Abbreviations: HR, hazard ratio; Klemera-Doubal Method Biological Age; PhenoAge, Phenotypic Age.

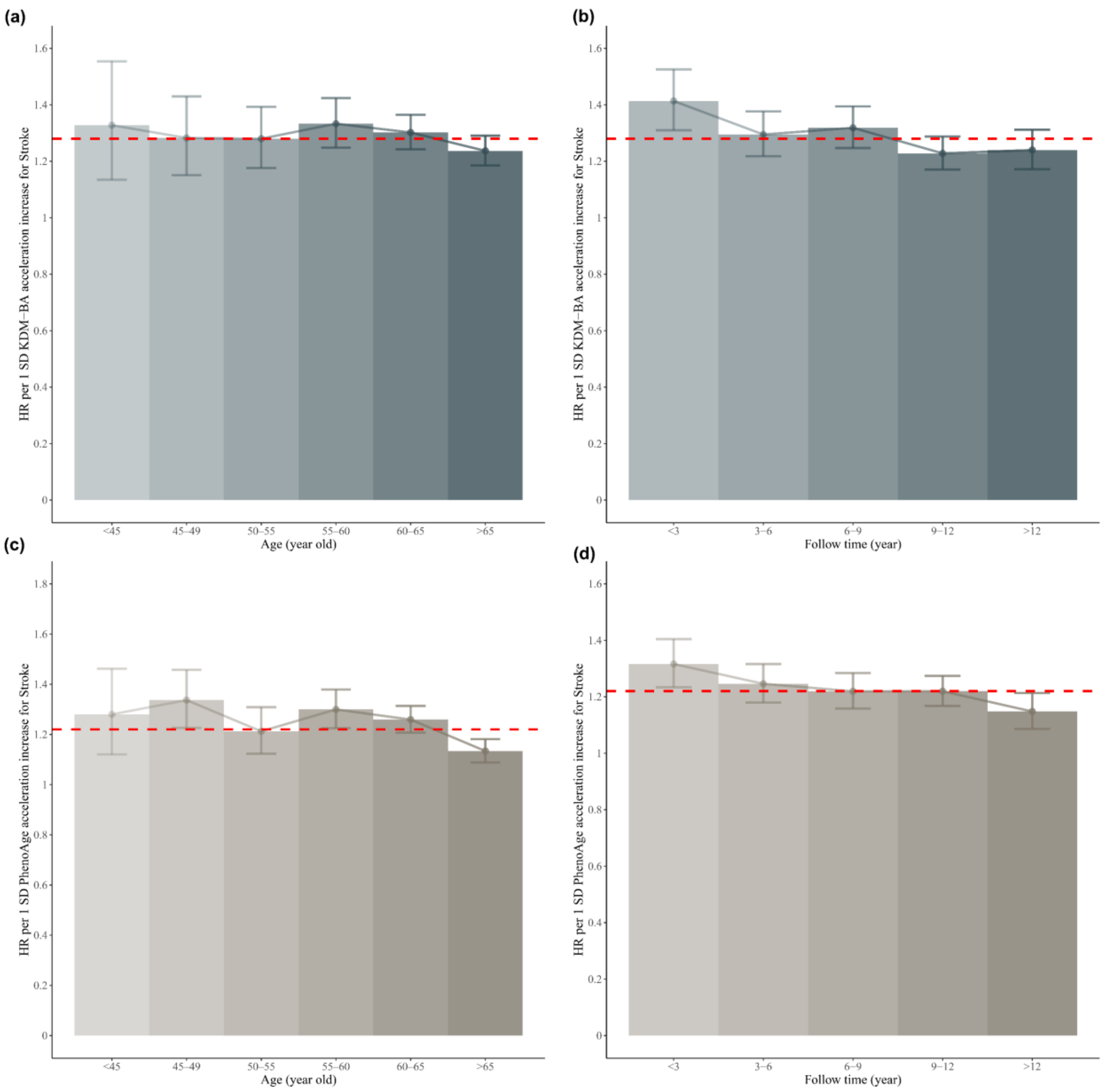

Figure S6. Association between biological age accelerations and risk of stroke using time-varying model with interaction terms between KDM-BA or PhenoAge acceleration and age (in 5-year intervals) (a and c) or between KDM-BA or PhenoAge acceleration and follow-up time (in 3-year intervals) (b and d).

Models for a and c adjusted for sex, assessment center, household income, years of education, employment status, Index of Multiple Deprivation, alcohol consumption, and behaviors score.

Models for b and d adjusted for age, sex, assessment center, household income, years of education, employment status, Index of Multiple Deprivation, alcohol consumption, and behaviors score.

Abbreviations: HR, hazard ratio; SD, standard deviation, Klemera-Doubal Method Biological Age; PhenoAge, Phenotypic Age.

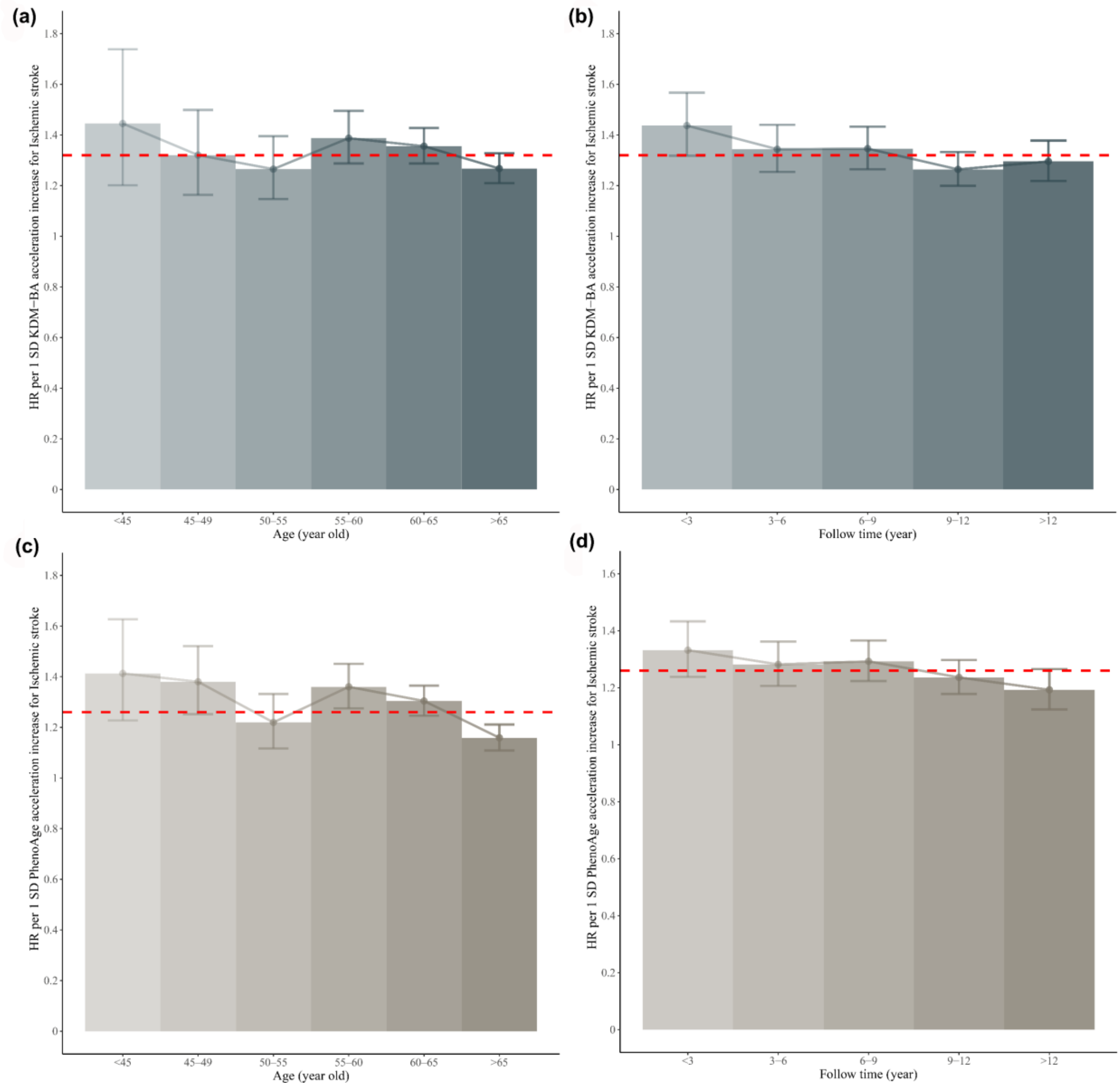

Figure S7. Association between biological age accelerations and risk of Ischemic stroke (IS) using time-varying model with interaction terms between KDM-BA or PhenoAge acceleration and age (in 5-year intervals) (a and c) or between KDM-BA or PhenoAge acceleration and follow-up time (in 3-year intervals) (b and d).

Models for a and c adjusted for sex, assessment center, household income, years of education, employment status, Index of Multiple Deprivation, alcohol consumption, and behaviors score.

Models for b and d adjusted for age, sex, assessment center, household income, years of education, employment status, Index of Multiple Deprivation, alcohol consumption, and behaviors score.

Abbreviations: HR, hazard ratio; SD, standard deviation, Klemera-Doubal Method Biological Age; PhenoAge, Phenotypic Age.

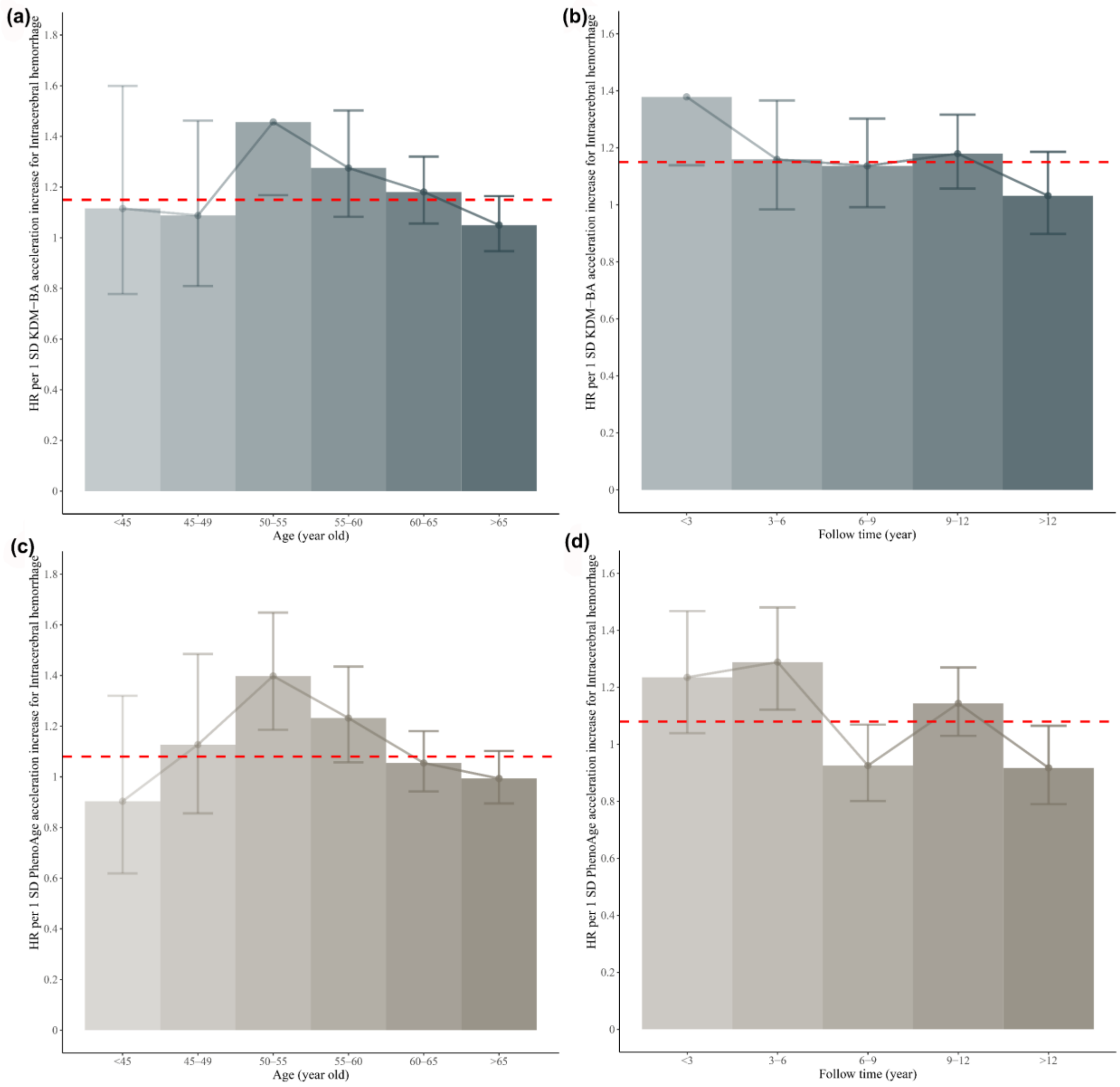

Figure S8. Association between biological age accelerations and risk of Intracerebral hemorrhage (ICH) using time-varying model with interaction terms between KDM-BA or PhenoAge acceleration and age (in 5-year intervals) (a and c) or between KDM-BA or PhenoAge acceleration and follow-up time (in 3-year intervals) (b and d).

Models for a and c adjusted for sex, assessment center, household income, years of education, employment status, Index of Multiple Deprivation, alcohol consumption, and behaviors score.

Models for b and d adjusted for age, sex, assessment center, household income, years of education, employment status, Index of Multiple Deprivation, alcohol consumption, and behaviors score.

Abbreviations: HR, hazard ratio; SD, standard deviation, Klemera-Doubal Method Biological Age; PhenoAge, Phenotypic Age.

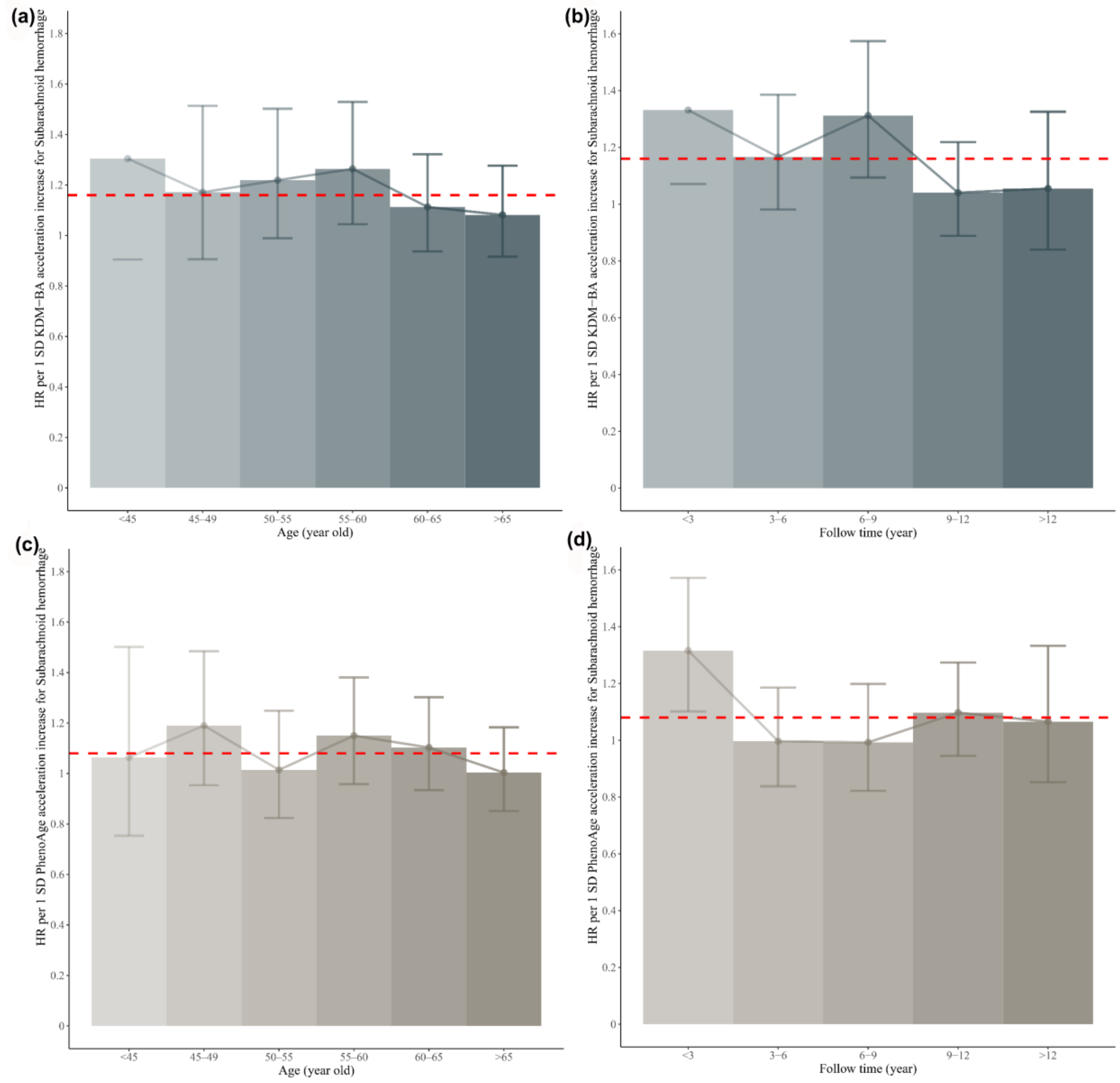

Figure S9. Association between biological age accelerations and risk of Subarachnoid hemorrhage (SAH) using time-varying model with interaction terms between KDM-BA or PhenoAge acceleration and age (in 5-year intervals) (a and c) or between KDM-BA or PhenoAge acceleration and follow-up time (in 3-year intervals) (b and d).

Models for a and c adjusted for sex, assessment center, household income, years of education, employment status, Index of Multiple Deprivation, alcohol consumption, and behaviors score.

Models for b and d adjusted for age, sex, assessment center, household income, years of education, employment status, Index of Multiple Deprivation, alcohol consumption, and behaviors score.

Abbreviations: HR, hazard ratio; SD, standard deviation, Klemera-Doubal Method Biological Age; PhenoAge, Phenotypic Age.
